# Statistical challenges in longitudinal microbiome data analysis

**DOI:** 10.1093/bib/bbac273

**Published:** 2022-07-14

**Authors:** Saritha Kodikara, Susan Ellul, Kim-Anh Lê Cao

**Affiliations:** Melbourne Integrative Genomics, School of Mathematics and Statistics, The University of Melbourne, Royal Parade, 3052, Victoria, Australia; Murdoch Children’s Research Institute and Department of Paediatrics, University of Melbourne, Bouverie Street, 3052, Victoria, Australia; Melbourne Integrative Genomics, School of Mathematics and Statistics, The University of Melbourne, Royal Parade, 3052, Victoria, Australia

**Keywords:** differential abundance, clustering, networks, compositionality, 16S, shotgun sequencing, relative abundance

## Abstract

The microbiome is a complex and dynamic community of microorganisms that co-exist interdependently within an ecosystem, and interact with its host or environment. Longitudinal studies can capture temporal variation within the microbiome to gain mechanistic insights into microbial systems; however, current statistical methods are limited due to the complex and inherent features of the data. We have identified three analytical objectives in longitudinal microbial studies: (1) differential abundance over time and between sample groups, demographic factors or clinical variables of interest; (2) clustering of microorganisms evolving concomitantly across time and (3) network modelling to identify temporal relationships between microorganisms. This review explores the strengths and limitations of current methods to fulfill these objectives, compares different methods in simulation and case studies for objectives (1) and (2), and highlights opportunities for further methodological developments. R tutorials are provided to reproduce the analyses conducted in this review.

## Introduction

The microbiome is defined as a collection of co-existing microorganisms within an environment, and is often viewed as a ‘mini-ecosystem’. The role of the microbiome in biological systems and in disease has been broadly explored [46, 54, 67], but is still lacking a thorough understanding of its structure and function [22]. In addition, temporal variation in a microbiome is inherently complex, with dynamic interactions between the host or environmental factors that are understudied so far [20, 68]. The goal of longitudinal studies is to address these issues [88].

We define longitudinal microbiome data as abundance data from individuals collected across multiple time points, similar to [14, 18]. The time dependency between measurements creates a correlation structure within individuals. Thus, longitudinal microbiome studies are designed to capture both within-subject dynamics and between-subject differences (heterogeneity among subjects) to address different analytical objectives. For example, microbiome researchers may want to identify microorganisms with differential abundance over time, between sample groups (e.g. case versus controls), between demographic factors (e.g. sex), or between clinical factors (e.g. mode of birth). Alternatively, they may be interested in identifying microorganisms evolving concomitantly across time and examining their temporal relationships between each other (i.e. biotic interactions). These biotic interactions can be in the form of positive interactions (e.g. cross-feeding of byproducts) or of negative interactions (e.g. competition for nutrients) [17]. Thus, the statistical analyses of longitudinal microbiome data correspond to three main objectives (1) to identify differentially expressed microorganisms; (2) to identify microorganisms evolving concomitantly across time and (3) to identify biotic interactions.

Although several methods have been proposed to address these objectives, the inherent complexity, sparsity, over-dispersed and high-dimensional aspects of longitudinal microbiome data are challenging, and require multivariate models with either specific data distributions (i.e. negative binomial distribution), or non-parametric models. Longitudinal studies also need to take into account repeated measurements with a temporal order, individual variability and variance that may change over time or differ for individuals in separate groups. A large number of missing time points in some individuals also pose a challenge for developing longitudinal microbiome methods.

Attempts have been made to address longitudinal microbiome data challenges [82]. However, most methods account for some but not all characteristics of microbiome data: they may ignore compositionality and struggle to jointly model over-dispersion and zero-inflation [31, 88], they might be unable to manage missing time points [8], or fail to capture the inter-dependency between microorganisms [88, 89].

Based on these three analytical objectives, we have reviewed existing statistical methods for longitudinal microbiome studies to highlight their strengths and limitations. We first describe the study design and the analytical challenges observed in a typical longitudinal microbiome study. We then review existing methods for each analytical objective and benchmark some of these methods in simulation and case studies. The R code to reproduce our analyses are available online.

## Data analysis of longitudinal microbiome studies

### Analytical objectives

**Figure 1:**
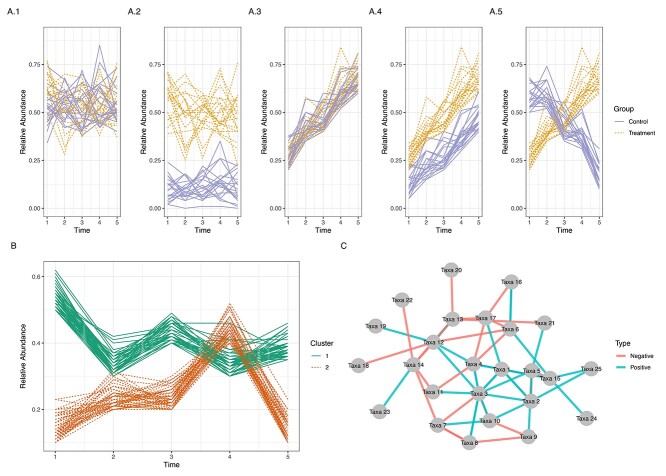
Three analytical objectives may arise from longitudinal microbiome studies: (**A**) To identify microorganisms with differential abundance over time, between groups or both between groups and over time. For example, a taxon’s abundance may not exhibit any between-group or temporal difference across subjects (A.1); may only exhibit group difference (A.2); or only time difference (A.3); both group and time differences without any interaction between the two effects (i.e., same slope with different intercept) (A.4); or with interaction. (**B**) To identify microorganisms with similar temporal patterns. For example taxa that belong to cluster 1 have a similar time trajectory, whereas other taxa that belong to cluster 2 exhibit a different pattern. (**C**) To understand biological and temporal relationships between different microorganisms. In this network representation, the positive (beneficial) interactions and negative (competitive) interactions are represented by blue and red colours, respectively. Note: in (A) each line represents a given taxon’s abundance for each subject, whereas in (B) and (C) each line or node represents a different taxon across groups of subjects.

A successful study begins with clear, well-defined scientific research objectives. We have identified common objectives of interest in longitudinal microbiome studies as summarized in Figure 1. The first objective is to study how microbial abundance changes over time between groups of interest (e.g. cases versus controls, disease or treatment groups, Figure 1A, and how the association between microbial abundance and other factors such as clinical outcomes, disease or treatments change over time [8]. In this context, both time and differences between patients or individual groups may be of interest. The second analytical objective is to cluster microorganisms with similar temporal patterns of abundance (Figure 1B). This analysis often requires us to model the temporal trajectories of each microorganism first. The third analytical objective is to construct a microbial network to understand the temporal relationships between sets of microorganisms (Figure 1C). The latter two objectives use methods based on distances, however, their goals and outputs differ. Clustering methods aim to partition taxa into clusters that reflect similar temporal behaviours, whereas network construction methods aim to discover positive or negative associations between a core set of taxa. Contrary to cluster visualizations that reveals similar taxa groups, most network visualizations reveal directed interactions between taxa and their strengths. Networks methods can include covariate information (e.g. age) as we discuss in this review, while currently available clustering methods are limited in this regard.

### Study design

**Figure 2:**
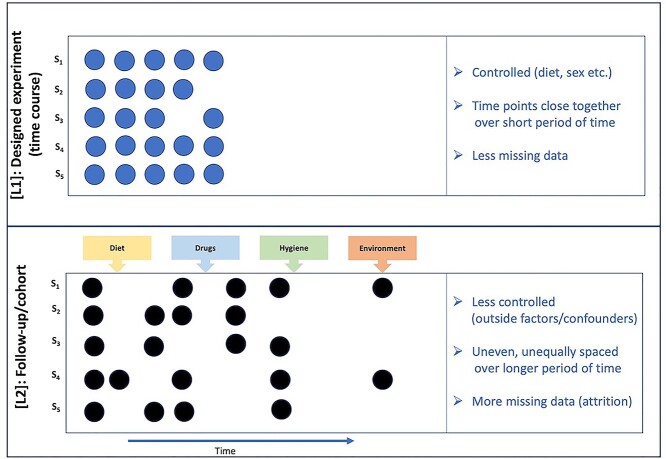
Characteristics of longitudinal microbiome studies. Data generated from a designed experiment [L1] – typically in mouse studies, include dense time points, with a similar number of time points for all subjects, whereas data generated from follow-up or cohort studies [L2] – typically in human studies, include uneven, unequally spaced time points for each subject.

Longitudinal microbiome data often arise from two different scenarios; from designed experiments (such as in mice) [L1] and from follow-up or cohort studies in humans [L2] (Figure 2). For longitudinal data of type L1, time points are typically close together than L2 studies with the same (or very similar) number of time points for each subject. In contrast, type L2 data typically feature uneven number of time points for subjects and unequally spaced time points, thus missing data due to attrition are more problematic in L2 than L1. Additionally in L2 studies, a number of external factors, such as diet, may also influence the microbiome but may often be unmeasured or uncontrolled, making the modelling more challenging.

### Data characteristics

Both L1 and L2 designs use 16S rRNA gene sequencing (16S) or metagenomic shotgun sequencing (shotgun) to generate raw sequenced reads. 16S sequencing is a form of amplicon sequencing that targets and reads a region of the 16S gene exclusively found in bacteria and archaea. Shotgun is an untargeted sequencing method that extracts all genomic material for microbial community classifications and gene annotations. From a statistical perspective, the data obtained from 16S and shotgun sequencing can both be represented as count tables containing the number of sequences per sample for a taxon [7]. In addition, from shotgun sequencing, a gene pathway table can be obtained, providing the number of sequences matching a specific gene function. To account for varying depths of reads across samples, researchers usually normalize the data by converting the raw abundances into relative abundances. Data produced by both these sequencing methods have many analytical challenges, due to the nature of the microbiome data, which are sparse, over-dispersed, of high dimension, multi-collinear, multivariate and highly variable [10, 23, 44, 65, 78]. Because of these analytical challenges, conventional methods for standard longitudinal data cannot be directly applied to longitudinal microbiome data [88], as we describe next.

#### Time trend and within-subject correlation

Compared to single time point data, longitudinal microbiome data include the presence or absence of trend over time. A trend, loosely speaking, is a pattern that shows the behaviour of the series as increasing or decreasing over a long period [52]. Since samples in longitudinal studies are collected over time, the ordering of the samples is inherent and irreversible and thus exhibit time-dependencies that are a function of time [20]. Ignoring the ordering of data points and the continuity of changes across time in the statistical analysis can lead to erroneous conclusions [21]. To model trends in longitudinal microbiome studies, spline models [69] or linear mixed models (LMMs) that regress the observations as a function of time [86, 88] can be used. These models can easily account for missing values observed across time through interpolation.

However, as samples are collected from the same subject for multiple times, we also need to consider auto-correlation among within-subject samples [36], where the independent error assumption in standard LMMs is no longer applicable. Dependent within-subjects errors in LMMs [86, 88] in the form of auto-regressive of order 1 (AR(1)) or continuous-time auto-regressive of order 1 can be used. A generalized Dirichlet-Multinomial distribution model [81] has also been proposed as an alternative approach to account for within-subject correlations [36].

#### Sparsity

Microbiome data from both single time point and longitudinal studies are sparse with frequently observed zero values, which is known as zero-inflation [34, 79]. These zeros can be due to (1) physical absence (a microorganism is not present at a particular time point), (2) undersampling of the microbial population or (3) sequencing error. In contrast to single time point microbiome data, where the degree of sparsity is typically microorganism-specific (proportions of zeros may vary across microorganisms), in longitudinal microbiome data the degree of sparsity is either microorganism-specific or time-specific (proportions of zeros in microorganisms may vary across time). To manage the sparsity issue, approaches such as zero-inflated beta regression models [8] have been proposed.

#### Over-dispersion and high inter-subject variability

In both single time point and longitudinal microbiome studies, data are over-dispersed, i.e. highly variable. Poisson models assume an equal mean and variance and lack flexibility to model over-dispersed data [58]. Therefore, negative binomial models [55, 80] have been extended to a longitudinal setting using a separate parameter to model the dispersion in the data [88].

High inter-subject variability of microbiome data is well known in single time point studies [1, 30, 85] but is exacerbated in longitudinal studies, as we need to consider difference of inter-subject variability across different time points. Thus, modelling approaches must consider this complexity, as well as the influence of measured and unmeasured confounders.

#### Multivariate and high-dimensional

These characteristics pertain to both single time points and longitudinal studies. The number of taxa included in a typical study may vary in the thousands [41, 84]. These microorganisms work in concert to modulate and influence their environment [38]. We need to consider the multivariate relationship among microorganisms at a single time point, as well as across time points in the case of longitudinal studies. However, most existing modelling approaches consider one microorganism at a time and fail to capture the multivariate nature of the data [8, 31, 69, 88]. Moreover, in a longitudinal setting, representing the interactions and similarities or connectivity between microorganisms becomes complex when the data are high-dimensional, resulting in high computational cost and low prediction accuracy of most statistical methods.

#### Compositional nature

**Figure 3:**
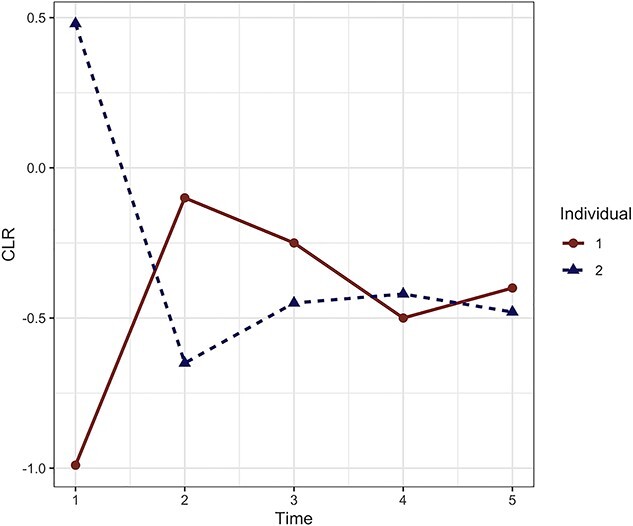
CLR trajectories for two individuals over five time points for a given taxon. The CLR is calculated per time point and per individual (i.e. per sample). For individual 1, the CLR abundance is greater than individual 2 at time 2, 3 and 5. However, we may not observe the same trend if we had access to the (true) absolute counts, as the geometric means are not directly comparable between samples. Similar problems arise when we consider multiple time points to assess differential abundance between two groups over time or between different time points within the same group.

Single time point microbiome count data are compositional due to unequal sequencing reads. This results in uneven library sizes across individuals. Longitudinal data add complexity as the compositionality arises from uneven library sizes across individuals *and* time points. In both experimental settings, each microorganism count is commonly converted into a relative abundance (proportion) in each sample, using ratio transformations, such as total sum scaling, producing compositional data. Thus, the data exist in a simplex. One pragmatic solution is to move from this simplex into a Euclidean space that is more suitable for current modelling methods. Several data transformations have been proposed, including centred log-ratio transformation (CLR). However, the interpretation of CLR transformed data should still be relative to other taxa as we refer to ratios of taxa to their geometric mean. The geometric mean can change if some taxa are removed beforehand. Thus, the CLR transformed data are incoherent to sub-compositions [56], as we illustrate in Figure 3. Note that prior to CLR transformation, an offset value is added to all raw counts to manage the zeros. However, this offset associated to CLR transformation will prevent from modelling zero counts.

**Figure 4:**
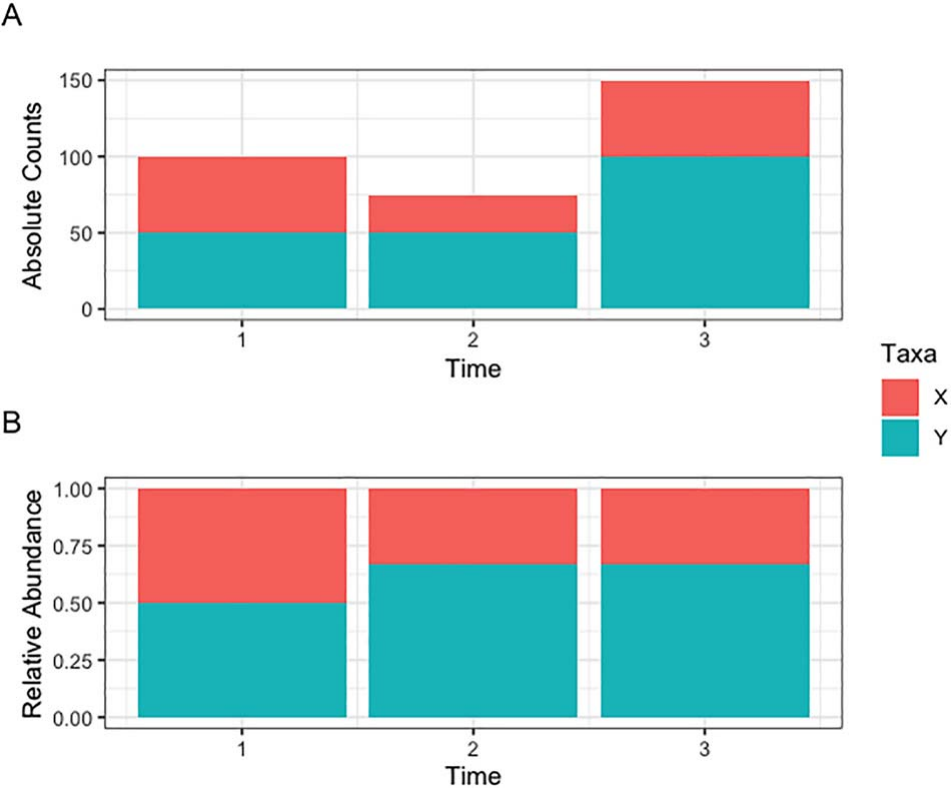
(**A**) For a given individual, we consider the true abundances (absolute counts) of two taxa (X and Y) across 3 time points and (**B**) their corresponding relative abundances. In (A), Y’s true abundance does not change from time 1 to time 2 but increases from time 2 to time 3. After time 1, Y’s true abundance is greater than X’s true abundance. In (B), after calculating relative abundance, both taxa have a relative abundance of 1/2 at time 1, but the relative abundance of X and Y changes to 1/3 and 2/3, respectively. Thus, we might incorrectly interpret that the abundance of Y increases from time 1 to time 2 and does not change from time 2 to time 3 (Figure adapted from [23] for a longitudinal scenario).

Compositionality may result in spurious correlations among taxa, as the presence or absence of one taxa depends on the others [56]. It is unclear whether the inflated correlations between taxa are due to normalization techniques such as proportional abundance or rarefaction (in complex microbial communities, such as the gut microbiome) or if it is due to having only a few dominant taxa (in low-diversity communities, such as the vaginal microbiome) [75]. In Figure 4, we extended the example discussed in [23] to multiple time points to illustrate that the relative abundance patterns observed in longitudinal microbiome data do not necessarily resemble the behaviour of the true abundance. Thus, an analyst should interpret the relative abundance results with regards to all other taxa and the library size of each sample (i.e. per individual and per time point).

Another question of interest is the identification of microbial signatures; i.e. groups of microbial taxa that are predictive of a phenotype of interest. However, we need to keep in mind the principles of compositional data analysis while answering this question. One such approach proposed for single time point analysis is *Selbal*, which is based on compositional balances — a measure that compares the average abundances of two groups of microbial species [60] based on their log ratios. *Selbal* is a forward selection approach that identifies the best subset of taxa according to their predictive power, measured by mean squared error or by the area under the receiver operating characteristic curve. However, the approach is not guaranteed to converge to a global optimum. Similar model selection approaches for longitudinal microbiome data are yet to be developed.

In the next sections, we review the strengths and limitations of methods currently available for longitudinal microbiome analysis according to the analysis objectives highlighted in Figure 1.

## Identification of microorganisms with differential abundance over time, between groups and between both group and time

Most approaches we have reviewed are univariate. They seek to identify differential microbial abundance over time, between groups of interest (e.g. disease or treatment groups), and between groups. These approaches fail to capture the complex interactions between microorganisms and may provide limited insight into the microbiome. We describe current methods that use either count data or relative abundance data for differential abundance analysis, and assess them in both simulation and case studies.

### Current methods

- Zero-inflated beta regression Model

Zero-inflated beta regression (ZIBR) simultaneously evaluates abundance changes over time and between groups for each taxon [8]. The model is applied to relative abundance (proportion) data and captures both the presence or absence of a microorganism – using a Bernoulli distribution, and non-zero abundance – using a Beta distribution. Thus, ZIBR can be considered as a mixture of a logistic regression and a Beta regression component. In order to capture within-subject correlations, individual-specific random intercepts are included to the model. Parameters of the model are estimated using Gauss-Hermite quadrature maximum likelihood. A likelihood ratio test is used to assess the effect of a covariate of interest (time, treatment or other clinical variables) on the presence or absence of a microorganism and its non-zero abundance. Time and its interaction with a given covariate can be incorporated into the model. Thus, ZIBR is highly flexible and can evaluate all effects illustrated in Figure 1A.

One of the strengths of ZIBR is its ability to account for the sparse nature of the data via use of the logistic component. However, ZIBR has several limitations. The model does not account for within-subject correlation structure (i.e. auto-regressive correlation structure) explicitly in the model [86]. The authors suggested that individual-specific random intercepts were often adequate in practice to capture these correlations. ZIBR cannot handle missing data at a given time point for a given subject [26, 74]. The method is also challenged when there are too few, or too many, zero values, which affect the accuracy of the logistic and beta component respectively [9]. It is also unclear how ZIBR manages compositional data. The authors argue that the *sum to one* constraint is not relevant as differential taxa are determined via use of a false discovery rate (FDR) and since each taxon is analyzed separately [8]. Other limitations highlighted by [45] include the lack of cross-part correlation in the model, which specifies the correlation between the individual-specific random intercepts in the logistic and beta components. This may result in inaccurate inference on treatment effect, as the magnitude of the relative abundance tends to be larger than expected for taxa that are more present, or dominant.

The method has been validated in simulation studies and applied to detect differential abundance between treatments for inflammatory bowel disease over an 8 week period [8], and subsequently in other microbiome studies [13, 24, 71].

- Negative binomial mixed model

Negative binomial mixed model (NBMM) was developed to detect associations between microbial counts (without transformation) and covariates (such as treatment, phenotype, age, dietary habits, etc.) while considering time trends of microbial abundance within and between subjects [88]. The iterated weighted least squares (IWLS, [87]) algorithm was extended to account for within-subject correlation structures. The algorithm iteratively approximate the NBMM using a LMM. NBMM model can be fitted with different fixed effects such as time effect, treatment effect, and interaction between time and treatment. Thus, similar to ZIBR, NBMM can also evaluate all effects illustrated in Figure 1A.

NBMM is able to handle over-dispersion with a dispersion parameter, as well as varying read lengths, by including an offset for differing total sequence reads. Fixed and random effects of varying types can be included, allowing for the inclusion of, or the adjustment for, confounders. A key strength is that NBMM can accommodate for differing correlation structures among observations from the same subject, compared to ZIBR that does not offer such flexibility. With adequate sample sizes, modelling of non-linear trends is also possible. However, the method does not explicitly handle zero-inflation.

NBMM has been validated in simulation studies and applied to previously published data [15] to detect differential abundance between term and preterm during pregnancy [88]. Of note, other authors had previously applied a zero-inflated negative binomial mixed-effects model (ZINBMM) in a longitudinal study of the vaginal microbiota during pregnancy [63].

- Block Bootstrap Method

Block bootstrap method (BBM) is an extension of a bootstrap method for longitudinal microbiome count data. The method uses a ‘moving block bootstrap’ approach using a blocking and re-sampling procedure while accounting for autocorrelation within the time series. To obtain bootstrap samples that approximate the distribution of the chosen statistic, blocks of temporally contiguous observations are constructed and resampled with replacement. An overlapping block bootstrap procedure was proposed to account for the small number of repeated observations [31], a procedure that has been demonstrated to produce the smallest mean squared error for most statistics [37]. To identify the optimal block size, a modified empirical sub-sampling approach was proposed. BBM aims to identify differential abundance between sample groups but does not focus on time effect. Thus, this method can only identify taxa that are differentially expressed between groups, as illustrated in Figure 1 A.2. *P*-values are adjusted for multiple testing using the Benjamini and Hochberg’s procedure [3] to rank the microbial variables.

BBM is nonparametric and does not require particular data distribution. It can handle within-subject dependency and accounts for unequal library sizes to address the compositional nature of the data. The authors report a high true positive and small false positive rate. As limitations, the method is computationally intensive and requires an adequate number of time points (at least five) to specify two tuning parameters (initial block size, number of repeat observations for sub-sampling). Sparsity and variability are still problematic and require pre-filtering to remove unwanted noise due to temporal variation (of both technical and biological origins). BBM does not account for other covariates and performs best when there is an equal number of observations for all subjects. In terms of interpretation, the method assesses whether the abundance is greater or lesser in one sample group compared to another but without quantifying this difference.

The method was validated in synthetic data, but has not been widely used in the literature so far. The authors applied BBM in three pregnancy studies to identify microbial variables differentially abundant between preterm versus term birth and whether these variables may overlap between studies, and in an oral microbiome study.

- SplinectomeR

SplinectomeR is an R package that uses weighted local polynomials (*loess* splines) to summarize data for hypothesis testing in longitudinal studies. *Loess* splines are suitable for microbiome data, as the data differ from classical models or shapes [69]. This framework includes three methods: the first method tests for the overall difference between two groups over the full time course, the second method tests for the difference between two groups at defined intervals to identify regions of time with group differences, and the third method tests for an overall trend in a single population over time. SplinectomeR can only assess group and time effects separately, and not their interactions as illustrated in Figure 1 A.5.

The methods in SplinectomeR are easy to interpret and can compare observations across multiple time points directly, without averaging or summarizing these points. They can also handle missing or unbalanced data. However, the methods may be influenced by outliers, particularly in sparse data sets, and do not account for compositional data. In addition, the third method that tests for overall trends can be time consuming as it is based on a permutation test.

The method was tested and validated on simulated data that included 10 individuals with 12 time points. The response variable was perturbed at three magnitudes in one or two regions of the time series and was detected by the third method. Using the prodigious data set [83], the authors evaluated the claims made on the dynamics of the gut microbiome over the first three years of life using the SplinectomeR methods. These methods were also applied to assess the effect of chemotherapy on the gut microbiota in acute leukaemia patients [59] and to test sex bias in gut microbiome transmission in newly paired marmosets [90].

- Zero-inflated Gaussian mixed models

Zero-inflated Gaussian mixed models (ZIGMM) was developed to account for within-subject correlations and other properties of microbiome data [86]. Similar to all methods presented above, ZIGMM is also univariate. However, the model allows to either use counts or relative abundances as input. Relative abundance are transformed using square root arcsine. Count data are transformed using pseudo counts log base 2. The transformed data are then modeled with a zero-inflated Gaussian distribution. Thus, ZIGMM is a mixture of a logistic regression and a Gaussian regression component. ZIGMM is fitted using an Expectation-Maximization (EM) algorithm using the standard procedure for fitting LMMs. Through simulations, the authors showed that ZIGMM outperformed various previously developed methods while being computationally efficient compared with the other two zero-inflated methods, ZIBR and ZINBMM. Similar to ZIBR and NBMM, ZIGMM can also evaluate time effect, group effect and time $\times $ group interaction effect as illustrated in Figure 1 A.

The main strength of ZIGMM is its ability to model time-dependent effects and correlations among samples within subjects. Additionally, the approach can include various types of fixed effects and random effects with both normal distribution and zero-inflated models. The method can also account for different auto-regressive correlation structures among samples, for example, AR(1) or continuous-time auto-regressive of order 1. Finally, the proposed method can analyze microbiome proportional data as well as count data generated from either 16S rRNA or metagenome shotgun sequencing technologies. However, ZIGMM also encountered the fitting issue of controlling false positive rates, specifically when complex data (including metagenomics) are being analyzed.

The method has been applied to two published data sets to detect associations between host covariates and taxa composition [63, 77]. The authors assessed the performance of ZIGMM in analyzing 16S rRNA data (raw counts) and shotgun sequencing data (proportions). ZIGMM was found to detect more vaginal bacteria taxa than LMMs and NBMMs. However in real data applications, the authors could not compare the performance of ZIGMM to ZIBR due to ZIBR’s inability to handle missing values.

- Bayesian semi-parametric generalized linear model

This multivariate approach considers succession change in taxa abundance with covarying physical or biological factors [39]. A model-based normalization is used to fit taxa counts. The normalization and estimation of the covariate effects on abundance is simultaneously carried out in a joint analysis of all taxa. When including time, group and their interactions as covariates in the model, one can evaluate all effects illustrated in Figure 1.

The method uses regularising priors with mean constraints [42] to avoid identifiability issues, and borrows information across microbial variables, samples and time points. Sparse estimates are produced, which is beneficial given the high dimension of the data and the high correlations between covariates. In contrast to other methods, this approach is multivariate. However, more developments are needed to flexibly capture differing shapes in response functions, incorporate variable selection and to allow for time-dependent covariates. Because of data sparsity, posterior computations must be handled with caution, and prior information need to be incorporated for accurate inference.

The performance of the model was assessed through a simulation study and applied to an ocean microbiome data set. The performance was found to be superior to the frequentist NBMM univariate model from [87]. Since then however, the NBMM method has been extended for longitudinal data (as listed above). A new extension has been proposed to include variable selection using asymmetric nonlocal priors regression coefficients [70]. This new approach is referred to as ‘Bayesian Sparse Multivariate regression’ [39].

- Fast zero-inflated negative binomial mixed model

Fast zero-inflated negative binomial mixed model (FZINBMM) models count data and is fitted using an EM-IWLS algorithm [89]. This method can evaluate time and group effects and time $\times $ group interaction effect as illustrated in Figure 1A. FZINBMM inherits features from LMMs, such as incorporating various types of fixed and random effects and within-subject correlations. The method also takes sparsity and over-dispersion of count data into account. Through simulations and real data, the authors showed that FZINBMM outperformed other count methods such as LMMs, NBMMs and ZIGMMs in terms of empirical power and high proportion of detected taxa. However, when the data were not highly sparse, FZINBMM performed similarly to ZIGMMs and NBMMs. Similar to other methods discussed in this section, FZINBMM also analyses one taxon at a time, and thus fails to capture the multivariate nature of microbiome data. Additionally, data compositionality is not taken into consideration.

**Table 1:** Summary of methods used to identify differential abundance over time, between groups and between group and time, in longitudinal microbiome studies. For each method, we specify the type of input data, the characteristics of microbiome data that are accounted for, and additional modelling features, such as inclusion of covariates. We specify the range of time points, individuals from the validation examples and the relevant R package name. In method ‘*SplinectomeR*’, users can define a minimum data sparseness threshold. The symbol $\checkmark $ for columns ‘Covariates’ and ‘Missing Values’ indicate methods that are suitable for analysing both L1 and L2 study designs. Others that do not satisfy the above condition are more suitable for analysing L1 study designs (L2 study design require methods that can handle confounders and missing values, Figure 2). RA= Relative abundance.

**Method**	**Input**	**Sparsity**	**Over-dispersion**	**Multi-variate**	**Composi-tionality**	**Co-variates**	**Missing Values**	**Explicit within subject correlations**	**Time points**	**Individuals**	**Package name**
									Simulation		
									5	50 - 150	
ZIBR	RA	$\checkmark $	$\checkmark $	-	$\checkmark $	$\checkmark $	-	-	Real data		ZIBR
									2 - 6	31 - 59	
									Simulation		
									5 - 10	50 - 150	
NBMM	Counts	-	$\checkmark $	-	-	$\checkmark $	$\checkmark $	$\checkmark $	Real data		NBZIMM
									52	40	
									Simulation		
									10- 15	20	
BBM	Counts	-	$\checkmark $	-	-	$\checkmark $	$\checkmark $	$\checkmark $	Real data		bootLong
									30 - 52	8 - 96	
									Simulation		
									12	10	
									R data		
SplinectomeR	RA	$\checkmark $	-	-	-	-	$\checkmark $	-	12	50	SplinectomeR
									Real data		
									12 - 28	16 - 39	
									Simulation		
	Counts,								5	50 - 150	
ZIGMM	RA	$\checkmark $	$\checkmark $	-	-	$\checkmark $	$\checkmark $	$\checkmark $	Real data		NBZIMM
									2 - 24	54 - 98	
									Simulation		
									55	3	
Bayesian semi- parametric generalized linear model	Counts	$\checkmark $	$\checkmark $	$\checkmark $	-	$\checkmark $	$\checkmark $	$\checkmark $	Real data		SBJReg
									55	2 - 3	
									Simulation		
									5	30 - 100	
FZINBMM	Counts	$\checkmark $	$\checkmark $	-	-	$\checkmark $	$\checkmark $	$\checkmark $	Real data		NBZIMM
									4	90 - 212	

FZINBMM was applied to two 16S rRNA and whole-genome shotgun sequencing count data sets [61, 62, 76]. The method was applied to detect the dynamic associations between taxa compositions, between different groups (i.e. use of antibiotics in infants, birth delivery type of mothers). The authors confirmed that the taxa they identified were biologically relevant as reported in the original study [61].

### Assessment on simulation data

In this section, we simulated data to assess the performance of differential abundance methods. The simulated data are similar to an L1 study in Figure 2 with no missing values to accommodate ZIBR. All taxa profiles had at least one zero count between time 0 and time 10 for at least one individual to accommodate ZIGMM and FZINBMM. The accuracy of the methods was assessed using sensitivity and specificity values.

#### Simulation design

**Figure 5:**
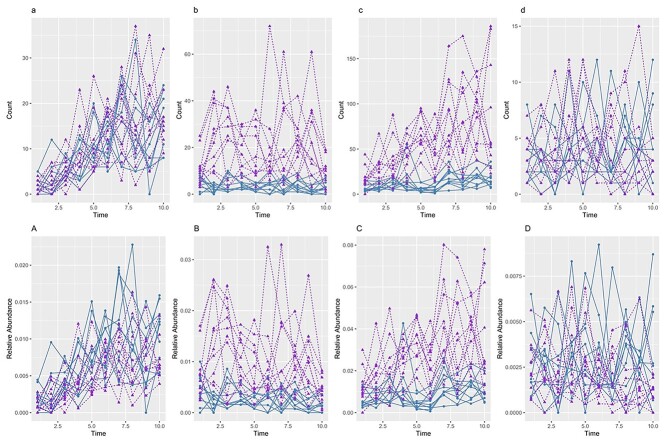
**Simulation study.** An example of simulated taxa with time, group, group $\times $ time and no effects with 0.3 dispersion and 0.2 AR parameters. The top panel shows the count data, and the bottom panel shows the relative abundance data for the same profiles (calculated by dividing the taxa count for a given individual at a specific time point by the total number of counts across all 300 taxa for that same time point and individual). Taxa count across time with a) time effect only, b) group effect only, c) group $\times $ time interaction effect and d) no effect. Relative abundances across time with **A**) time effect only, **B**) group effect only, **C**) group $\times $ time interaction effect and **D**) no effect.

We simulated longitudinal count data from a generalized linear model with a negative binomial distribution using the ‘tscount’ R package [43]. More details on the simulation strategy are provided in the Supplemental Section S1. We estimated realistic parameter values for dispersion and auto-regressive (AR) coefficients using the pregnancy data from [15]. Nine case scenarios were created with three dispersion parameters (i.e. noise) and three AR values (see Table 2). For each case scenario, 50 data sets were simulated with 300 longitudinal profiles (referred to as taxa) measured on 10 time points and 20 individuals from two groups. The 300 profiles included ten profiles in each category that were differentially abundant through:

- time only,
- group only,
- time, group and their interaction,

and the remaining profiles that were not differentially abundant (i.e. noise). Figure 5 illustrates five simulated profiles with different effects with 0.3 dispersion and 0.2 AR.

#### Simulation results

We present the simulation results for moderate parameter values (i.e. dispersion=0.3 and AR=0.2) for all methods reviewed earlier, with the exception of BBM that was deemed computationally expensive, and the Bayesian semi-parametric generalized linear model, which is not conducive to sensitivity and specificity measures as it does not generate *P*-values. All R packages used are listed in Table 1.

**Table 2:** **Simulation study.** Parameter settings for nine simulation scenarios with three dispersion parameters and three AR parameters. The dispersion parameter is used to control the noise among different individuals, whereas the AR parameter is used to control the within-subject correlations for an individual. For each of these nine scenarios, 50 data sets were simulated with 300 longitudinal taxa profiles measured on 10 time points and 20 individuals from two groups. The 300 taxa profiles were further divided into time, group, interaction effects, as indicated. The parameter values used in the linear predictors of the generalized linear model are the intercept, AR coefficient and covariate coefficients (see Supplemental Section S1). The intercept for each individual was generated from a uniform (0, 5) distribution in these models and beta values used for each taxa profile are indicated in the table. The choice in parameter values is detailed in Supplemental Section S1.1

Dispersion/ AR Parameter		0.04; 0.2; 0.4			
0.1; 0.3; 0.6	**Effect**	**Number of taxa**	**Time**	**Group**	Group*Time
	Time	10	1.5	0	0
	Group	10	0	13	0
	Group+Time+Group*Time	10	1.5	13	5
	Noise	270	0	0	0

**Figure 6:**
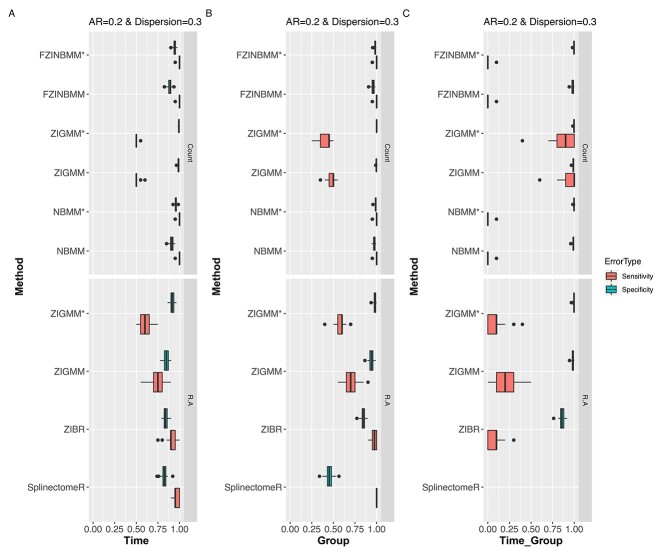
Simulation study. Sensitivity and specificity results from differential abundance methods for time effect, group effect and time $\times $ group interaction effect (AR = 0.2, dispersion = 0.3). Each figure is partitioned in to two panels according to input type (i.e. count or relative abundance). NBMM and FZINBMM with an AR within-subject correlation structure correctly identified taxa with time or group effects compared to other methods. The ZIGMM count model outperformed other methods in identifying time and group interaction effects. Overall, the count methods performed well in detecting time, group, and time and group interaction effects. ‘*’ indicates a model fitted with AR structure for within-subject correlation; As a reminder, SplinectomeR tests for time and group effects separately in contrast to other methods where time, group and time and group interactions are included in the same model.

Figure 6 shows the sensitivity and specificity results for time, group, and time $\times $ group interaction effects. With the exception of ZIGMM, all other methods based on count data (NBMM, FZINBMM) performed well in detecting the time effect and group effect. However, the ZIGMM count model outperformed all methods to detect variables with time and group interaction effects. Among methods based on relative data (e.g. ZIGMM, SplinectomeR), ZIBR performed well in detecting both time and group effects.

As expected, sensitivity was higher when dispersion was low. Conversely, specificity was low when dispersion was high (Supplemental Figures S10, S11, S12). When comparing the effect of the three AR parameters, we observed that specificity was improved for the models with increasing AR within-subject correlation structure compared to the models without the within-subject correlation structure. Thus, ignoring the AR structure may generate spurious effects over time, especially for data with a high within-subject correlation (i.e. high AR parameter) (Supplemental Figures S12). However, the improvement in specificity with the AR parameter is at the expense of lower sensitivity.

### VREfm case study

We applied all methods to a longitudinal study investigating the role of the gut microbiome during Vancomycin-resistant *Enterococcus faecium* (VREfm) colonization following an antibiotic treatment [51]. The authors used 16S rRNA sequencing to profile the bacterial community composition in 9 mice over a 14-day period. The mice were administered a ceftriaxone antibiotic treatment at days 6 and 7, and were then colonized with VREfm at day 9 (Supplemental Figure S4). We considered the samples during the naive phase of the experiment (day 0 to day 5) as control group, and the samples during the VRE phase of the experiment (day 9 to day 14) as treatment group (see details in Supplemental Section S3). After filtering, 193 taxa were tested for time effect, group effect and their interaction effects with the same methods as in previous Section. With the exception of ZIBR and SplinectomeR, all other methods resulted in at least one error during model fitting due to the methods’ technical constraints (as detailed in Supplemental Section S3.3). Using a significance level of 0.05, we reported the number of significant taxa with time, group and time $\times $ group interaction effects for each method along with the number of common significant taxa across different methods. Significant taxa with time, group and time $\times $ group interaction effects varied widely across methods (see Supplemental Figures S6, S5 and S7). Overall, we found that FZINBMM without the AR within-subject correlation produced the most number of significant taxa across all effects, a result that we might expect given the large antibiotic and VREfm effects compared to the naive phase.

**Figure 7:**
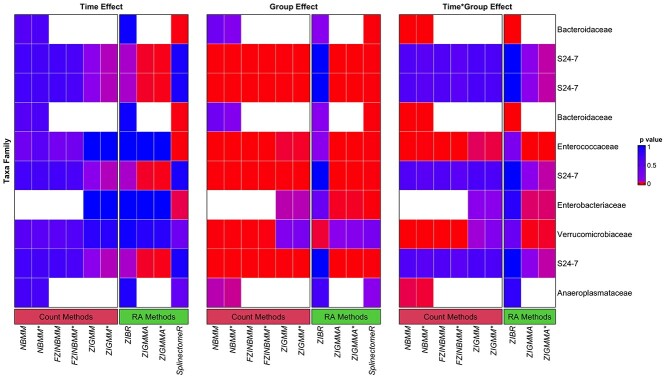
VREfm study. P-values for time, group and their interaction effects for the ten most abundant taxa. P-values range from < 0.05 (red) to 1 (blue). White cells indicate methods that produced a numerical error. The most prominent effect in these taxa is the group effect, as expected. We observed group differences in *Bacteroidales*, *Enterococcaceae* and *Enterobacteriaceae* families. Significance level = 0.05, ‘*’ indicates a model fitted with a AR within-subject correlation structure.

In Figure 7, we explored the most abundant taxa with statistically significant effects across all effects. A majority of these taxa were from *S24-7* family (i.e. currently known as *Muribaculaceae* family) or *Bacteroidaceae* family which belongs to the *Bacteroidia* class. Figure 7 indicates that the taxa from *Bacteroidia* class had strong group difference. According to the authors of the study [51], *Bacteroidia* class were of biological relevance as their dominance in the naive phase was shifted in response to ceftriaxone antibiotic treatment and regained in the late phase of the experiment. Since the treatment group was colonized with VREfm pathogen that belongs to the *Enterococcus* genus, we expected a group difference in the *Enterococcaceae* family. All methods except ZIBR output significant *P*-values for group and interaction effects for *Enterococcaceae family*. In addition, one taxon belonging to the *Enterobacteriaceae* family with a group and a time $\times $ group interaction effects have been found to be associated with the antibiotic-mediated disruption of the microbiota [35].

## Identification of microorganisms with similar temporal patterns

Clustering methods mainly seek to identify microorganisms that evolve similarly across time. We review the applications of such approaches for longitudinal microbiome data and briefly discuss other clustering methods with slightly different analytical objectives. Similar to the previous section, we assess the methods in both simulation and case studies.

### Current methods

- Dynamic time warping distances

A dynamic time warping (DTW) distance-based clustering method was proposed to identify taxonomic groups with similar temporal patterns [2]. Data normalization is crucial in this context to minimize the differences caused by different orders of magnitude in taxa abundances, using for example proportion or rarefaction normalization. The DTW distance between two time series is then calculated and normalized by the average sum of the absolute difference between each time series and its vertically flipped image (i.e. mirror image), resulting in a value called ‘Time-DTW distance’ that is bounded between zero and one. The distances among different taxa are visualized using a heatmap, and then hierarchically clustered based on their similarity measures and taxonomic hierarchies. Compared to Euclidean distance based clustering methods, DTW takes into account the distortion across time series, and is thus suitable to identify temporal behaviours that are out of phase [2].

- Partitioning around medoids and agglomerative clustering

In [12], the authors applied these two types of clustering algorithms to microbiome time-series data. Partitioning around medoids (PAM) [33] is a popular algorithm for implementing k-medoids clustering [53], whereby data are allocated into k clusters (similar to k-means clustering). In k-medoids clustering, each cluster is represented by a cluster medoid that is most centrally located in the cluster. A medoid is a data point that minimizes the average dissimilarity between itself and all the other data points in the cluster. Because PAM uses medoids instead of means, the approach is less sensitive to noise and outliers, compared to k-means clustering [32].

Agglomerative clustering (Hclust) is a hierarchical clustering algorithm which adopts a *bottom-up* approach to group taxa based on their similarity. The algorithm starts by assigning each taxon to a singleton cluster and then iteratively merges pairs of clusters with the highest similarity until all clusters have been merged into a single cluster. The authors used the complete linkage distance for clustering, which tends to produce compact clusters as the distance between two clusters is defined as the maximum value of all pairwise distances between the taxa in the two clusters. For both clustering approaches, variance-stabilization and scaling normalization of the data were applied.

- Clustering using principal component analysis and sparse principal component analysis

Multivariate dimension reduction techniques principal component analysis (PCA) and sparse principal component analysis (sPCA) were used to cluster taxa profiles with similar temporal patterns [5]. In this approach, the original data matrix with $N$ number of biological samples, $P$ number of taxa and $T$ number of time points are first summarized with linear mixed model splines (LMMS), resulting in a spline fitted matrix of size ($T \times P$). The matrix dimensions are then further reduced by PCA, resulting in $H$ principal components of length $T$ and their associated loading vectors of length $P$. For a given dimension of PCA, strongly correlated profiles are identified through their loading coefficients and their signs (i.e. positive or negative). To determine the optimal number of principal components, $H$, the average silhouette coefficient [64] is used. With sPCA, only a subset of the taxa profiles are selected. Different subsets define each of the components, where the selected taxa are highly correlated within a component. Thus, sPCA does not highlight profiles that deviate from the average cluster profile.

These approaches are most suitable when the number of time points is small (i.e. 5–10) and when the data are expected to follow regular and similar trends across time [5].

In addition to the methods presented above, other clustering methods were applied for different analytical objectives. For example, two distinct clustering approaches (PAM and Hclust) were used to group samples to discover comparable microbiome states between different individuals [19].The Ananke algorithm was proposed to cluster distinct temporal patterns in microbiome sequence data (i.e. FASTA-formatted data) rather than sequence-identity-based taxa [27]. As another example, k-medoids clustering was also used to cluster time points based on Jenson—Shannon divergence matrix [2]. Thus, clustering methods can be applied to longitudinal microbiome data to answer a wide range of biological questions.

### Assessment on simulation data

In this section, we simulated data to assess the performance of clustering methods. We first needed to summarize the profiles across different subjects to reduce subject-level variability. This can be done by calculating the mean or median at each time point for each taxon (i.e. mean or median profiles) or by using smoothing splines and more sophisticated LMMS as presented with the PCA approach. LMMS were shown to outperform the mean, median and smoothing splines profiles to identify temporary changes [72].

#### Simulation design

**Figure 8:**
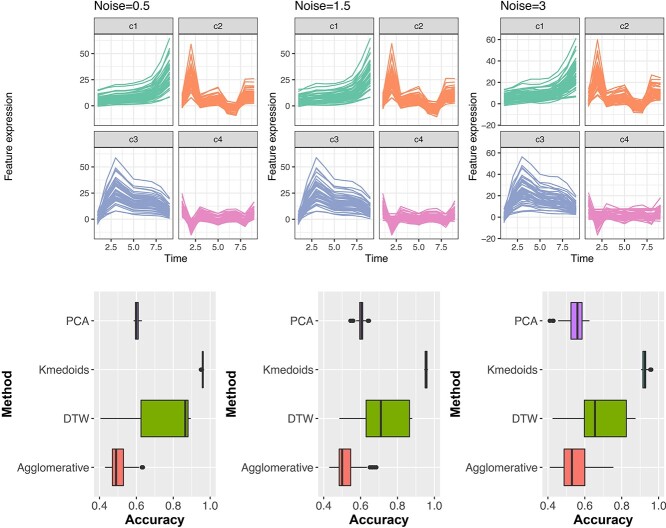
Simulation study. Clustering accuracy for PCA, k-medoid, DTW and Agglomerative clustering for original LMMS profiles. The top panel provides an example of the time profiles for different noise levels. The bottom panel shows that on average, k-medoid clustering method outperformed the other methods when clustering the LMMS profiles with varying noise levels.

We used the simulation from [5], which included profile modelling using LMMS. We generated 200 reference profiles from time one to time nine that belonged to four clusters (50 profiles each). These reference profiles were then used to simulate five new profiles (corresponding to five different individuals) with a fixed level of noise (see Supplemental Section S2 for details). Thus, for a given noise level, 100 data sets were generated with 200 time profiles and five individuals. Time profiles were then modeled with LMMS, resulting in 100 data sets of size ($9 \times 200$) for each level of noise. These LMMS profiles were then used as inputs in clustering methods. We calculated the clustering accuracy by dividing the number of correctly clustered profiles by the total number of time profiles. Three noise levels (0.5, 1.5, 3) were considered to assess the effect of inter-individual variability on clustering accuracy. We compared the original, centred, scaled, centred and scaled LMMS profiles as inputs in each of the clustering methods (see Figures 8 and Supplemental Figures S13, S14 and S15).

#### Simulation results

We present the simulation results for all clustering methods presented earlier. As DTW is implemented as a web application in [2], we used the ‘dtwclust’ R package [66] for more flexibility in our analyses. To assess PAM, agglomerative and PCA clustering, we used the ‘stats’ [57], ‘cluster’ [49], ‘mixOmics’ [16] and ‘timeOmics’ [4] R packages, respectively.

Across all three noise levels (i.e. 0.5, 1.5, 3), k-medoid clustering resulted in the highest median clustering accuracy compared to other clustering methods (i.e. PCA, DTW, Agglomerative clustering, bottom panel of Figure 8). However, as expected, the clustering accuracy across all approaches decreased as the noise levels increased. When the noise level increased, the number of straight line LMMS profiles also increased (top panel of Figure 8). Interestingly, we observed different clustering performances depending on whether the LMMS profiles were centred, scaled or centred and scaled. For example in Supplemental Figure S13, when the LMMS profiles were centred, both k-medoid clustering and PCA clustering had a similar clustering accuracy for the lowest noise level 0.5. However, for scaled LMMS profiles, k-medoid clustering outperformed all other methods across all noise levels (Figure S14). For the centred and scaled LMMS profiles, k-medoid clustering, DTW and agglomerative clustering all had similar median cluster accuracy, but DTW had a high variability in its accuracy (Supplemental Figure 15). With the highest noise level, all clustering methods performed similarly for centred and scaled LMMS profiles.

### VREfm case study

We compared the clustering methods on the VREfm case study described earlier and Supplemental Section S3. Clustering methods were applied separately to two groups: naive phase (control group) and VRE phase (treatment group). In the control group, we expect a stationary behaviour as the microbiome should be relatively stable during the naive phase. In the treatment group, we expect groups of taxa with increasing abundance with time as some microbial communities recover from the antibiotic effects, and groups of taxa with decreasing abundance with time as some microbial communities recover from VREfm colonization. This was confirmed in the clustering results in Supplemental Figure S8. Taxa clustered from the control samples resulted in mostly straight lines, whereas taxa clustered from treatment group resulted in clusters with increasing or decreasing abundance trends. In Supplemental Figure S9, we explored the most abundant taxa clustering for the treatment group. All four methods assigned the taxon related to VREfm (i.e., *Enterococcaceae*) in a small cluster indicating its difference from other taxa assigned to larger clusters. Additionally, two taxa belonging to the *Bacteroidaceae* family in treatment group showed an increased abundance with time, and were assigned to the same cluster by both PCA and DTW clustering, suggesting a good performance of these two approaches. However, these two taxa were assigned to two different clusters by k-medoids and agglomerative clustering. The increase in *Bacteroidaceae* family in treatment group is expected, as [51] demonstrated a shift in the dominance of *Bacteroidia* class in response to ceftriaxone treatment.

## Understanding biological and temporal relationships between microorganisms

Another prime analytical objective of longitudinal microbiome analysis is to study temporal relationships among taxa. These relationships can have a positive, negative or no impact on the taxa involved. Inferring the associations between taxa can be used to predict the effect of community alterations or perturbations. In this section, we review three strategies to identify temporal associations between microorganisms, either across all individuals, or per individual.

### Current methods

- Two-stage dynamic Bayesian Nnetwork

Dynamic Bayesian networks were employed to model the gut microbial ecosystem in infants using the CGBayesNets (Conditional Gaussian Bayesian Network) MATLAB software package [50]. This approach uses a simplified two-stage dynamic Bayesian network (TS-DBN) that assumes that the network model only depends on the variable values at the current time point and the previous time point. The other assumption is that all transitions of interest are from the prior time point to the subsequent time point and not within each time point. TS-DBN builds networks with discrete and continuous variables, where a conditional probability distribution is specified over discrete variables and a conditional linear Gaussian density function is defined over continuous variables. In the case of small sample sizes, the inclusion of clinical and demographic variables may result in over-fitting [50]. Currently, this method is limited to two time points and may not perform well for rare taxa, as network connections showed lower levels of confidence [50].

Using the same DBN approach, others developed a novel approach to infer causal relationships between microbial taxa, clinical conditions, and demographic factors [48]. The authors used spline estimation and DTW techniques to align microbial relative abundance data. The aligned time-series were then integrated across individuals to learn DBNs. In three different longitudinal microbiome studies, the authors showed that temporal alignments improved prediction performance compared to MTPLasso (described below) and TS-DBN.

- Granger causality based interaction networks

The network model in the web application ‘TIME’ [2] is based on Granger causality [2] that assesses pairwise causality between two taxa ‘A’ and ‘B’, for a given individual. The principle of this statistical test is that if taxon A affects taxon B, then the past values of A must have some information that would not be available otherwise about the future values of B. The influence of A on B is ascertained by comparing the goodness of fit between two regression models. In the first regression model, the present values of B are regressed on the past values of both taxa. In the second regression model, the present values of B are regressed on its own past values. If the former regression results in significant increase in the goodness of fit compared to the latter, then A is said to ‘Granger cause’ B, but causality should not be confused with correlation.

In addition to the pairwise Granger causality, the method also identifies potential causal relationships among all taxa using ‘Granger Lasso Causality’ [28]. LASSO is a $L_1$-norm penalty to the sum of squared errors and is used as a variable selection and regularization method in regression models [73]. One can use both Granger approaches (i.e. pairwise Granger causality and Granger Lasso Causality) to select causal pairs and generate a directed causality network. However, causal interactions in these networks are statistical predictions that do not explain the causality (interactions could be due to an indirect causes). Thus, interpretations should be made with caution. Incorporating other functional data such as metabolic co-dependencies could strengthen interpretation [40]. One limitation of this method is that it does not take clinical or demographic variables into consideration when building the interaction network.

- Microbial time-series prior Lasso

Microbial time-series prior Lasso (MTPLasso) was developed to infer interactions between microorganisms [47]. Similar to Granger causality based interaction networks, this method is also used to develop individual-specific networks.

First, count data are transformed into relative abundances. Dynamics and interactions amongst microorganisms are modelled using the discrete-time Lotka-Volterra (LV) model for population dynamics [29]. A stochastic noise component is added to the LV model to account for the affect of environments factors and other noise sources (i.e. measurement errors) on the abundance change. Assuming that the LV system of equations have a unique steady-state solution and that the noise component follows a normal distribution, the LV model is converted to a standard linear regression problem, with LASSO penalization to select the interactions that minimize the mean square error. However, since the data are high-dimensional with a limited amount of time points, the number of possible interactions between microorganisms is far greater than the number of time points. As a consequence, the regression problem becomes intractable. To solve this issue, Hofbauer et al. [29] integrated biological information obtained from the scientific literature and metagenomics data sets. The model performance and robustness were enhanced through bootstrap aggregating [6] and re-ranking [25]. Similar to the previous method, MTPLasso also ignores any clinical or demographic variables when modelling the interaction network.

The network methods discussed in this section widely differ in their analytical objectives and input requirements. For example, Granger causality-based interaction networks and MTPLasso aim to fit a network model explaining the relationship between taxa for each subject, whereas TS-DBN aims to fit a network model explaining the relationship between taxa for the entire data set. Although Granger causality-based interaction networks and MTPLasso have the same analytical objective they require different inputs. MTPLasso requires biological information from scientific literature and metagenomic datasets to solve the regressions, whereas Granger causality-based interaction networks only require the abundance values observed. As such, we were unable to compare these approaches on data.

## Conclusion

Longitudinal microbiome studies can help in understanding the links between the microbiome and health or disease. In this review, we categorized longitudinal analysis methods according to three main research objectives: (1) differential abundance over time, between groups and between both group and time, (2) temporal pattern clustering and (3) network modelling. We highlighted the current limitations of existing method to manage the inherent characteristics of microbiome data (i.e. compositionality, sparsity, over-dispersion, high within-subject variability).

Differential abundance and clustering methods’ main limitation is the compositional nature of the data. By ignoring compositionality, these methods may produce biased or misleading results. In addition, the majority of differential abundance methods are univariate, and thus ignore the mutual relationships between microorganisms, which may lead to spurious results. It is important to note that zero-inflated models such as ZIGMM and FZINBMM should be used for taxa with excessive number of zeros. In practice, however, researchers may prefer to fit a specific method for all taxa regardless of their degree of sparsity. Therefore, zero-inflated models could be improved with greater flexibility to include or exclude the zero-inflated part of each taxon based on their degree of sparsity.

Network models are promising for longitudinal microbiome data analysis, but are still at their infancy. These models infer interactions between microorganisms to understand the role and influence of microorganisms in diseases, and their co-evolution with time. Another line of analysis is to investigate changes of microbial networks across time (for example due to antibiotic intervention). Future promising applications of networks models will be the designs of synthetic microbiome to validate data-driven ecological networks [11]. However, substantial methodological developments are still needed to establish causal inference networks. As we highlighted in our review, there is also a lack of common analytical objective in these approaches. For example, some models focus on individual-specific networks while others focus on a full sample-specific networks.

To conclude, we have identified a growing need for flexible approaches that can suitably handle the intrinsic challenges of microbiome longitudinal data. The ultimate goal of these approaches is to capture the complex interactions, dynamics and influences of microorganisms that can shed light into the mechanisms underpinning health and disease. If successful, these methods have a potential to advance preventive, personalized and predictive medicine.

**Key Points**

- Longitudinal microbiome studies are conducted to understand the temporal variations of the microbiome, which is inherently complex, with dynamic interactions between microorganisms, host and environment factors.
- Longitudinal microbiome data have inherent data characteristics that are common to both microbiome data (such as compositionality, sparsity and over-dispersion) and longitudinal data (such as within-subject correlation, high-variability between time points).
- We identified three analysis objectives, ranging from differential abundance analysis, to clustering and network modelling.
- Most methods developed to address one of the three objectives do not take the data characteristics into account, which may lead to biased or spurious results, and lack flexibility in their applications.
- Methods for longitudinal microbiome data are still at their infancy, and require substantial methodological developed to understand biological and temporal relationships between microorganisms.

## Data Availability Statement

All analyses were conducted using the R software and are fully reproducible using the codes available on the gitHub link https://github.com/SarithaKodikara/Longitudinal_microbiome_data_analysis.

## Supplementary Material

Supplemental_bbac273

## Author Biographies

**Saritha Kodikara** is a research fellow in Computational Genomics & Statistics. She is interested in combining statistical modelling and computational frameworks for microbiome studies.

**Susan Ellul** is a PhD student at the Clinical Epidemiology and Biostatistics Unit at the Murdoch Children Research Institute, Melbourne.

**Kim-Anh Lê Cao** is associate professor in Statistical Genomics. Her laboratory focuses on the development of computational and integrative multivariate methods for feature selection in biological data.
