# Supplementary material for: Statistical challenges in longitudinal microbiome data analysis
Source: Brief Bioinform. 2022 Jul 14;23(4):bbac273. doi: 10.1093/bib/bbac273 (PMC9294433; doi:10.1093/bib/bbac273)
Supplement: Supplemental_bbac273 [file supplemental_bbac273.pdf]

S1 Simulation design to assess differential abundance methods

In this section, we describe the details of the simulation study for comparing differential abundance methods.

Longitudinal count data $Y_t : t \in \mathbb{N}$ were simulated from a generalised linear model with a negative binomial distribution and a group covariate X, using the 'tscount' R package [4]. $X = 1$, if an individual belongs to the treatment group and zero otherwise (i.e., control). We used a first order auto-regressive (AR) component in the linear model to capture the within-subject correlation. The conditional mean $\mathbb{E}(Y_t|F_{t-1})$ of the count time series was modelled using $\{\lambda_t : t \in \mathbb{N}\}$, which depends on F_{t-1} – the history of the joint process $\{Y_{t-1}, t, X\}$. Thus, the linear model for λ_t is as follows:

$$\lambda_t = \beta_0 + \beta_1 Y_{t-1} + \eta_1 t + \eta_2 X + \eta_3 tX \tag{1}$$

where β_0 is the intercept, β_1 is the AR parameter, ϕ is the dispersion parameter,and η_1, η_2, η_3 are the covariate parameters (i.e., time, group, etc). Parameter values for β_0, β_1, η_1, η_2, η_3 and ϕ are indicated in Table 2.

A negative binomial distribution with mean λ_t and dispersion $\phi \in (0, \infty)$ was used to model $Y_t|F_{t-1}$:

$$Y_t|F_{t-1} \sim NegBin(\lambda_t, \phi) \tag{2}$$

We chose a negative binomial distribution rather than a Poisson distribution to model over-dispersed data.

In order to evaluate univariate methods, we had to assume that taxa are independent. Therefore, we simulated separate count time series for each taxon. However, we ensured that the 300 taxa had different taxa profiles. For example, 10 taxa only had a time effect and were generated with the same parameter setting given in Table 2. We considered *9* settings with *3* dispersion parameters (ϕ) and *3* AR parameters (β_1) that were chosen based on the estimated values from the pregnancy data from [3] in Equation 1. The reasons for choosing the parameters listed in Table 2 are explained in the next subsection.

S1.1 Realistic parameter values for the simulation design

To identify realistic values for AR and dispersion parameters, we fitted three generalised linear models for three microbial variables (e.g. Operational Taxonomic Units, OTUs) using the 'tscount' R package [4] on the pregnancy data from [3]. The original study collected data on OTUs across a *40*-day period, but we limited our parameter estimation on three individuals who had continuous observations from the *26*th to the *35*th day. This is to maximise the number of original samples (as illustrated in Figure S1) and to fit into a ten time-point period for our simulations. The estimated parameter values for pregnancy data are listed in Table S1.

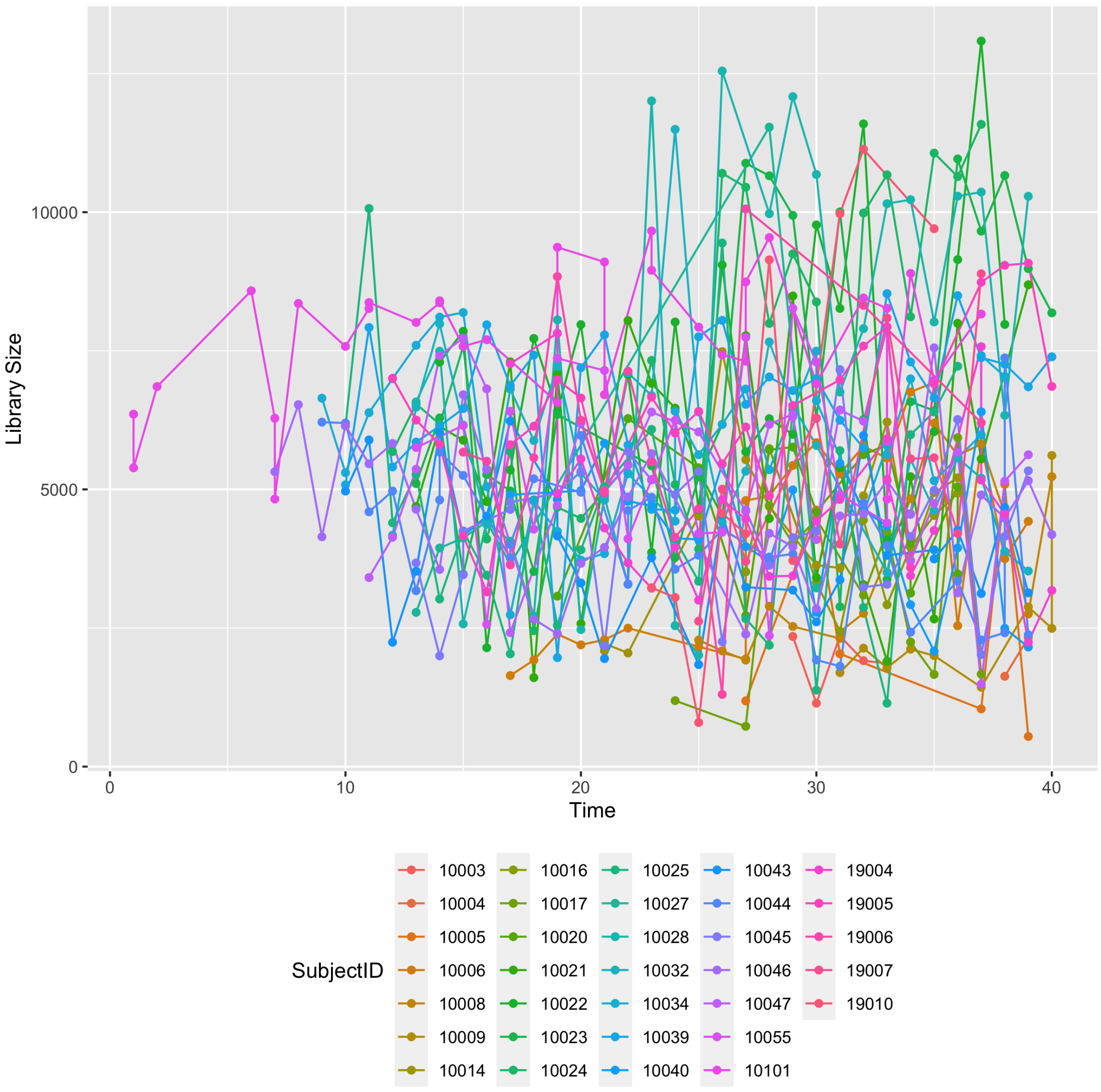

Figure S1: **Pregnancy study from [3].** Time series plot of library sizes for all individuals except seven with preterm pregnancies. We chose the time period of 26 to 35 days to estimate our parameters in our simulation study.

Table S1: **Pregnancy study from [3].** Estimated parameter values for AR and dispersion using the 'tsglm()' function for three OTUs observed in three individuals across time points 26 to 35.

OTU ID	Individual ID	Estimated AR parameter	Estimated Dispersion parameter
X4430843	10021	0.47	0.98
X933546	10039	0.04	1.10
X137183	10040	0.06	0.19

Based on Table S1, we chose 0.04 and 0.4 as the low and high values for the AR parameter. For the dispersion parameter, we chose 0.6 as the high value despite the fact that the data

had dispersion values close to one. This is to preserve the overall effects (i.e., increasing trend) observed across time against noise, as illustrated in Figure S2.

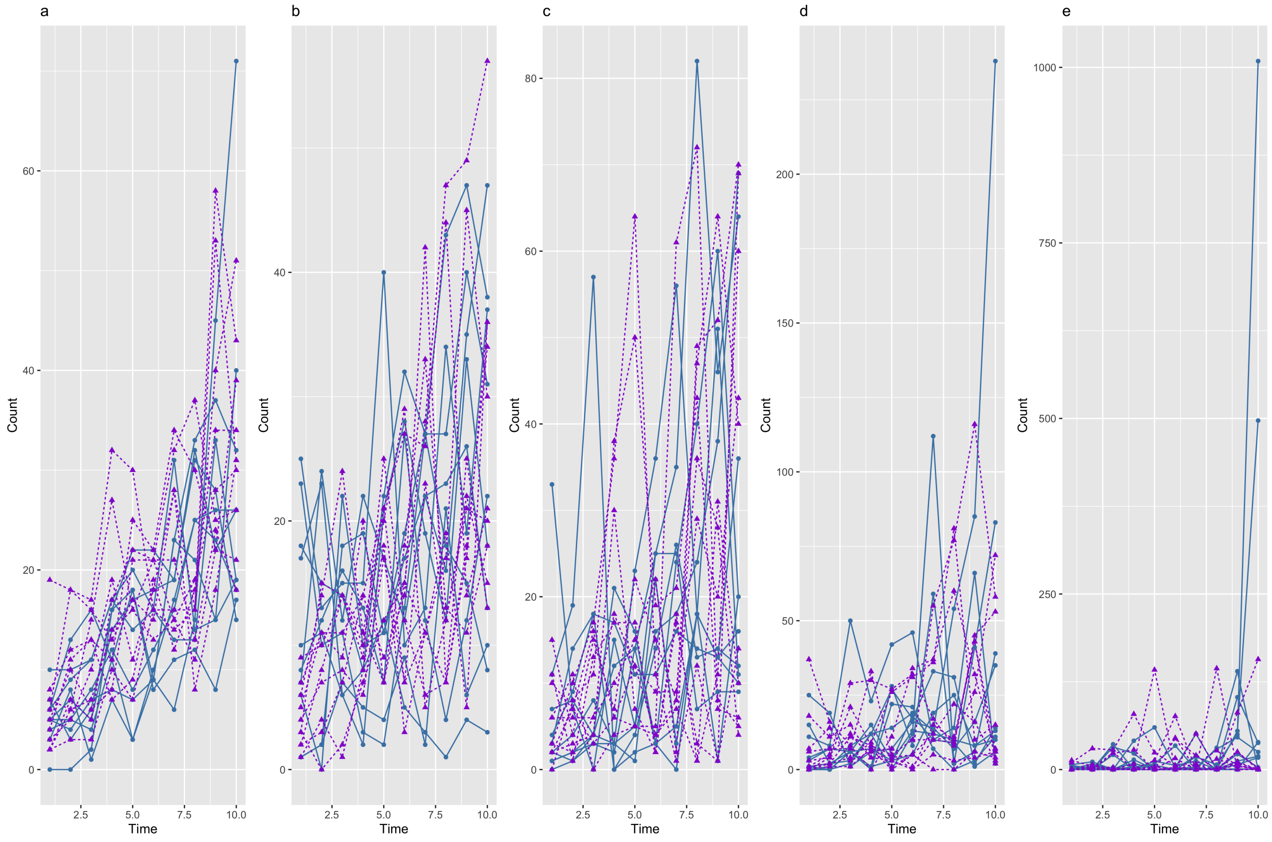

Figure S2: **Simulation study.** Example of simulated taxa with time effect (0.4 AR parameter) with respect to five different dispersion values a) 0.1, b) 0.3, c) 0.6, d) 1 and e) 5. As the dispersion parameter value increases, the increasing trend becomes less apparent, thus justifying a dispersion parameter of 0.6 in our simulations.

To further confirm our parameter choices, we also estimated the AR and dispersion in the VREfm study (control group only). Figure S3 shows that a choice of AR parameter values 0.04, 0.2 and 0.4 is reasonable as our estimates ranged from 0 to 0.6 in the real data. Similar to the pregnancy data, the estimated dispersion values had a high variability between zero and fifteen. Therefore, and as discussed earlier, we deemed a high value of 0.6 reasonable to preserve the effects of interest (time and group).

The parameters for time, group and time group interaction effects were selected based on visual inspection of the simulated data to obtain strong time, group and time $\times$ group effects.

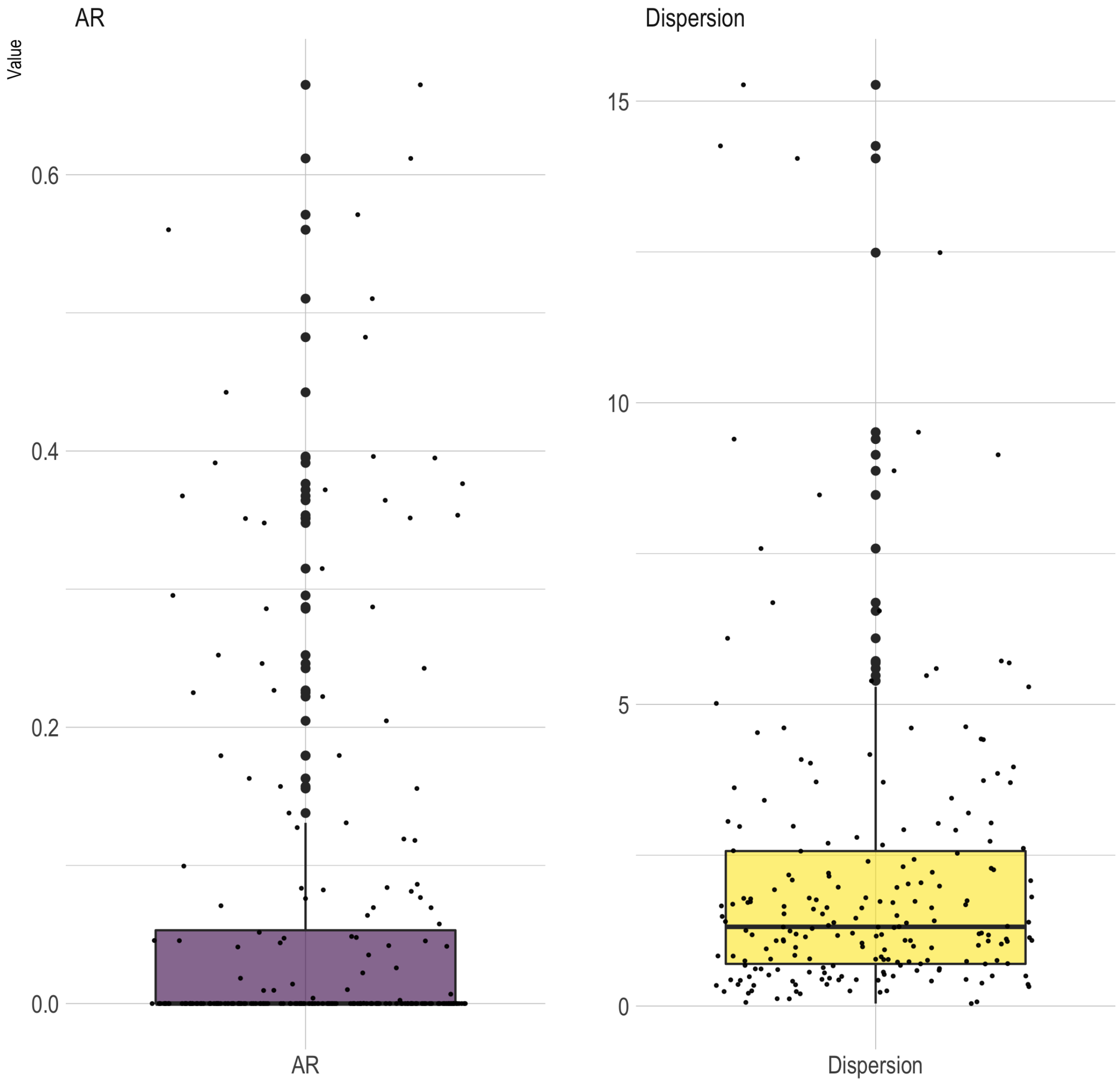

Figure S3: **VREfm study.** Box-plots representing the estimates for AR and dispersion parameters using the 'tsglm()' function for the control group. The parameter values used in our simulation for AR (i.e., 0.04, 0.2, 0.4) are within the range of the estimated AR parameter for this study.

S2 Simulation design to assess clustering methods

Using the same simulation setting as in [1], we first simulated 200 reference profiles $x^r = \{x^r(1), x^r(2), \ldots, x^r(9)\}$, $r = 1, \ldots, 200$, where each x^r is observed on *9* time points $t = 1, \ldots, 9$. Each of these reference profiles belonged to four different clusters, resulting in 50 time profiles for each cluster. To take into account some inter-individual variability, we generated 5 new profiles $x_i^r(t)$, $i = 1, \ldots, 5$, where each x_i^r is observed on 9 time points $t = 1, \ldots, 9$ and *200* profiles $r = 1, \ldots, 200$. For a given individual i, we simulated its observed value $x_i^r(t)$ at time t from a Gaussian distribution with mean $\mu = x^r(t)$ and variance σ^2. To vary the noise levels, we repeated the above simulation steps with $\sigma = 0.5, 1.5, 3$. The five

profiles (individuals) which were simulated in this way were then modeled with linear mixed model splines (LMMS) to summarise the time profiles across different individuals to reduce subject-level variability. The fitted values from LMMS were then used as inputs in clustering methods. For each noise level, we generated 100 of these LMMS data sets of size (9×200).

S3 VREfm case study

S3.1 Study design

Mu et al. [5] used 16S rRNA sequencing to capture the bacterial community composition in 9 mice that were administered a ceftriaxone antibiotic treatment across two days and were then colonized with Vancomycin-resistant Enterococcus faecium (VREfm) at a single time point. Mice were housed three cages in groups of five, and fecal samples were collected over a 14 day period from the same three mice. Figure S4 indicates the experiment phase across the time line along with the time points when the sample were collected.

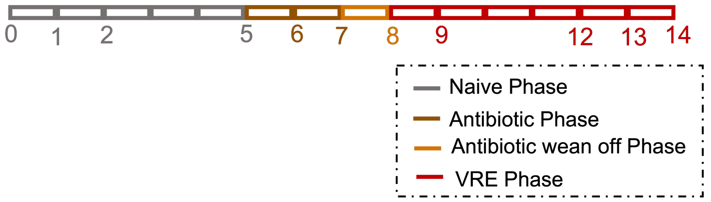

Figure S4: **VREfm study.** Phases of the experiment over the 14-day period and days where samples were collected for 16S rRNA gene sequencing. The mice were administered a ceftriaxone antibiotic treatment across days 6 and 7. On day 9 they were colonized with VREfm.

S3.2 Analysis design

To identify differentially abundant taxa between naive and VRE phases, we divided the data set into two groups based on the experiment phase. We considered the naive phase as the control group, and the VRE phase as the treatment group. Even though the same four mice appears in both groups, we considered the two phases as independent as the microbiome disruption following antibiotic treatment is strong. For both groups, we assumed the observations to be recorded from time 1 to time 4. The time alignment between both phases is imperfect but we assumed that it did not impact our analysis as we are studying the overall significance in time and group across several methods. The modified data set is referred as 'VREfm data' hereafter.

S3.3 Differential abundance results

The data contained 3,574 taxa across four time points (i.e., 1 to 4). After data filtering that removed taxa for which the sum of counts were below 0.01% compared to the total sum of all counts, 193 taxa remained. We applied the same methods as those in the simulation study to identify the differentially abundant taxa. Only ZIBR and SplinectomeR ran with no errors for all taxa. The highest number of errors were observed for FZINBMM and NMBB with an AR within-subject correlation structure (32 and 28 taxa, respectively). FZINBMM without an AR within-subject correlation resulted in 5 errors. NBMM without an AR within-subject correlation and all ZIGMM methods (i.e, count methods and relative abundance methods with and without AR component) resulted in 3 errors. This analysis thus highlights the potential lack of flexibility of these approaches when analysing real microbiome data.

We first investigated the taxa with a group effect. Figure S5 illustrates the number of significant taxa from each method along with the number of overlapping taxa using the upsetR package [2]. SplinectomeR resulted in a very high number of significant taxa compared to other methods (150 out of 193). Interestingly, our simulation results showed that SplinectomeR group effect had a low specificity (Figure 6 B) which may indicate that this method is sensitive to group difference. ZIGMM and ZIBR led to the lowest number of significant taxa compared to other methods. Our simulation results (Figure 6) indicated that ZIGMM had the lowest sensitivity results for the group effect. A small overlap of only six taxa was observed between all methods.

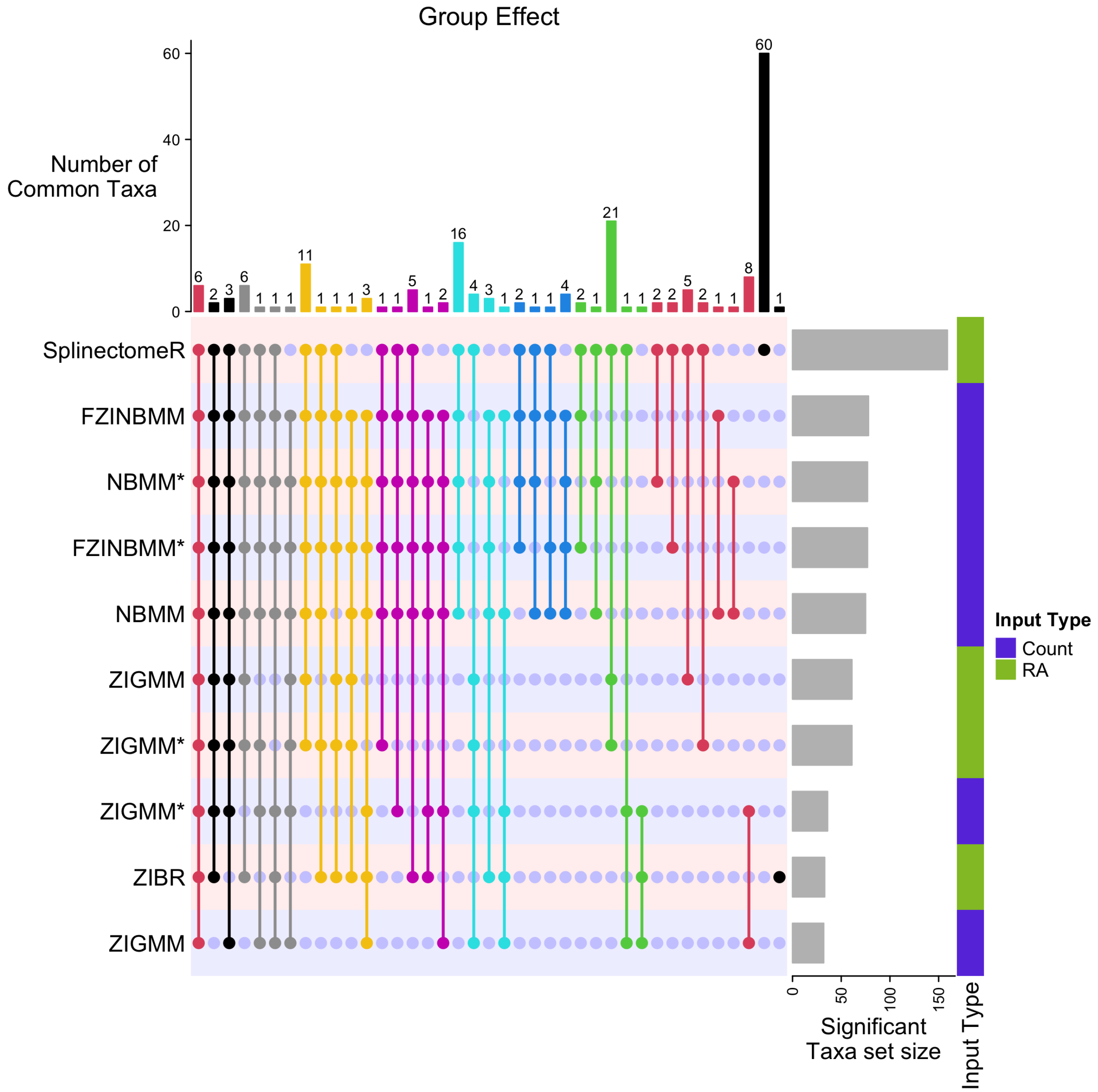

Figure S5: **VREfm study.** Number of taxa with a significant group effect for each differential abundance method and their overlap. SplinectomeR has the highest number of differentially abundant taxa , while ZIGMM count method without AR within-subject correlation has the lowest nuumber. Only six common taxa were declared significantly different between groups across all methods. '$*$' indicates a model fitted with AR structure for within-subject correlations

We then studied the time effect. SplinectomeR resulted in a very low number of significant taxa compared to other methods (Figure S6). SplinectomeR seeks for an overall trend regardless of the group type. As there is a high variability between groups in this dataset, the spline model is not able to capture the time effect accurately. All other methods identified 19 common taxa with significant time effect. Count methods resulted in a larger number of taxa with a significant time effect compared to relative abundance method. This finding aligns with the high sensitivity and specificity results we observed in count methods in our simulation results (Figure 6 A). In our simulation ZIGMM count method performed best in identifying the interaction effect (Figure 6 C). However, in this real data set, negative binomial models (i.e., FZINBMM, NBMM) resulted in the highest number of significant taxa with interaction effect (Figure S7, 32 taxa in common).

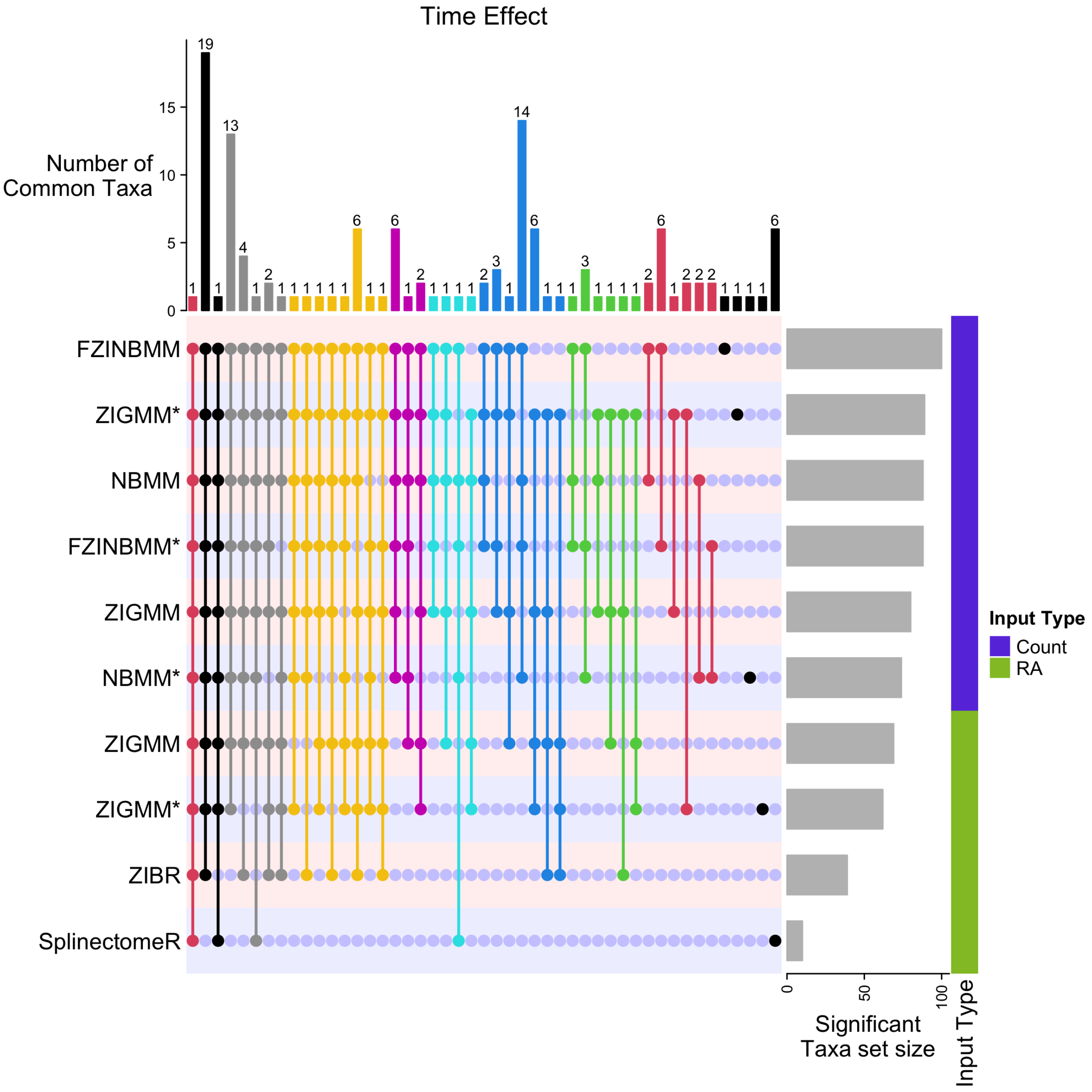

Figure S6: **VREfm study.** Number of taxa with a significant time effect for each differential abundance method and their overlap. Except for splinectomeR, all other methods identified 19 common taxa with significant time effect. Count method led to the highest number of significant taxa compared to relative abundance methods. '$*$' indicates a model fitted with AR structure for within-subject correlations

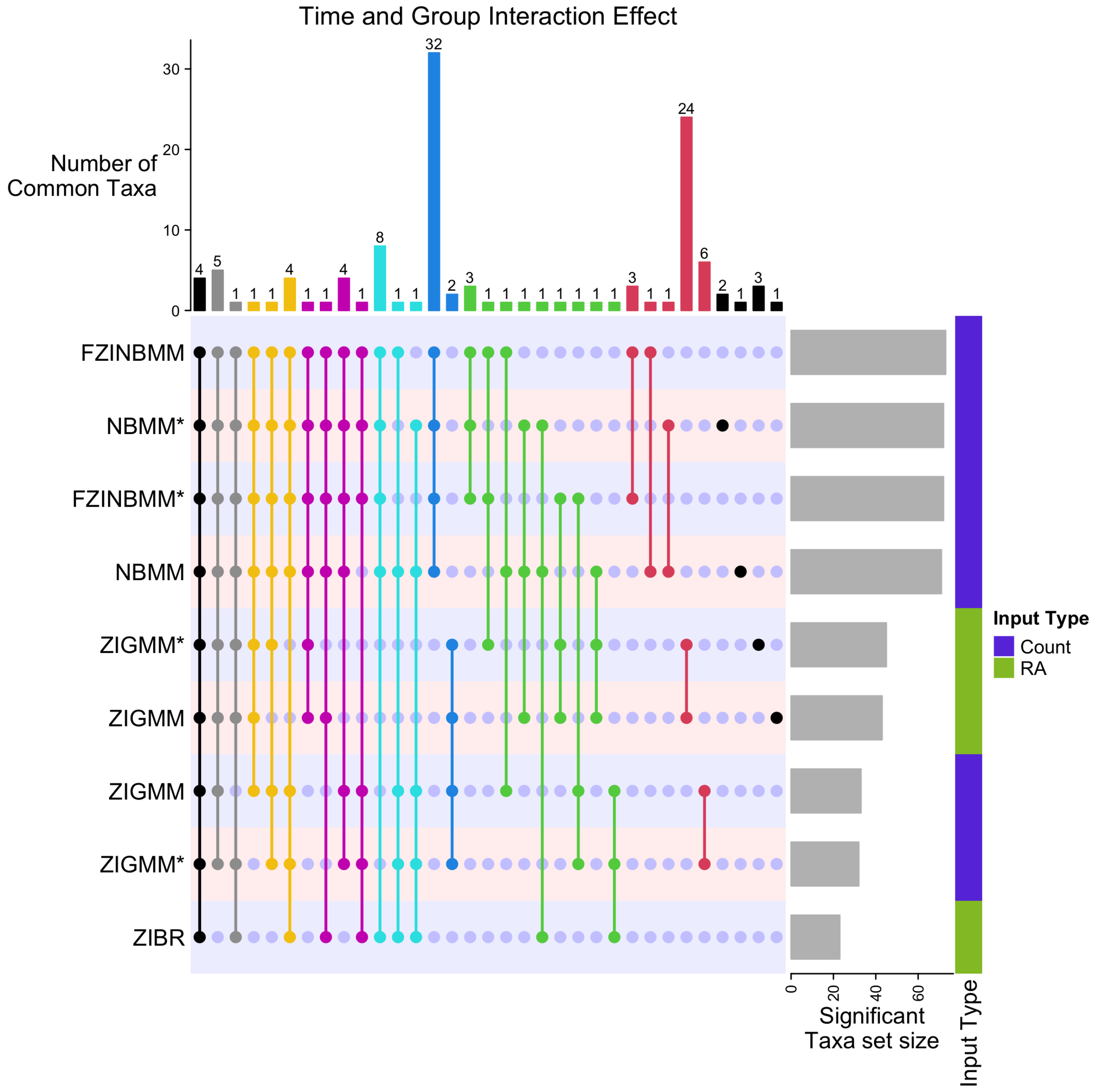

Figure S7: **VREfm study.** Number of taxa with a significant time and group interaction effec for each differential abundance method and their overlap. All negative binomial models led to the highest number of significant taxa with 32 taxa in common. '∗' indicates a model fitted with AR structure for within-subject correlations

S3.4 Clustering methods results

Figure S8 illustrates that the control group resulted in more straight lines compared to the treatment group. This is expected as the naive phase reflects a stable microbiome, whereas the VRE phase should highlight recovery of the microbiome from the antibiotic disruption and VRE colonization. Figure S9 explores the cluster assignment for top most abundant taxa in the treatment group. K-medoids and agglomerative clustering produced identical cluster assignments for the top most abundant taxa. All methods identified the taxa related to VREfm (*Enterococcaceae*) to be different from other taxa. Additionally, taxa that belong to the *Bacteroidaceae* family (Taxa_451 and Taxa_483) had a increasing abundance across time, and were assigned to the same cluster in both PCA and DTW clustering but were separated in

K-medoids and agglomerative clustering.

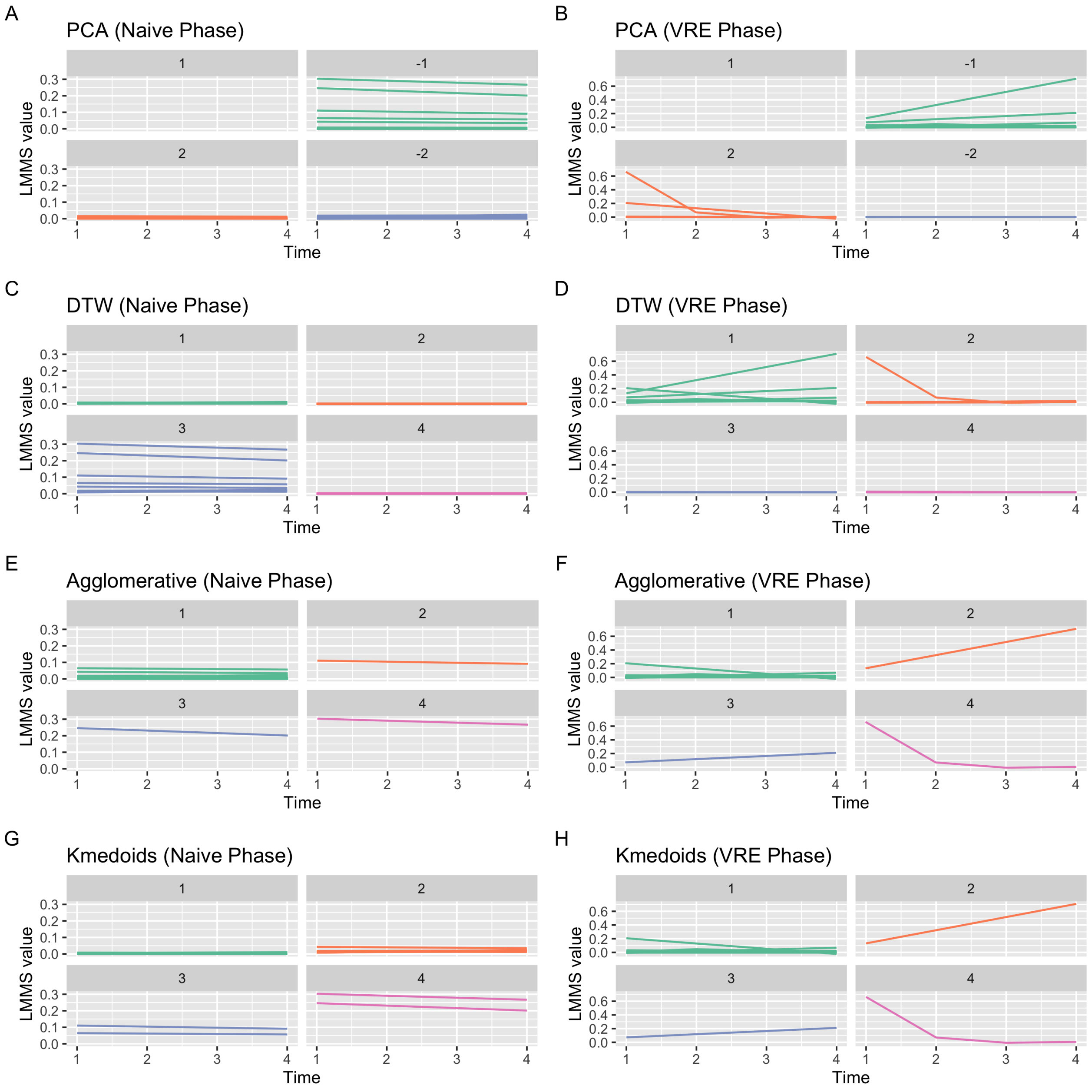

Figure S8: **VREfm study.** Cluster assignment using PCA, K-medoid, DTW and Agglomerative clustering for controls and treatment group. Each LMMS taxa profile is shown across time. As expected, the control group results in stable microbiome compared to the treatment group.

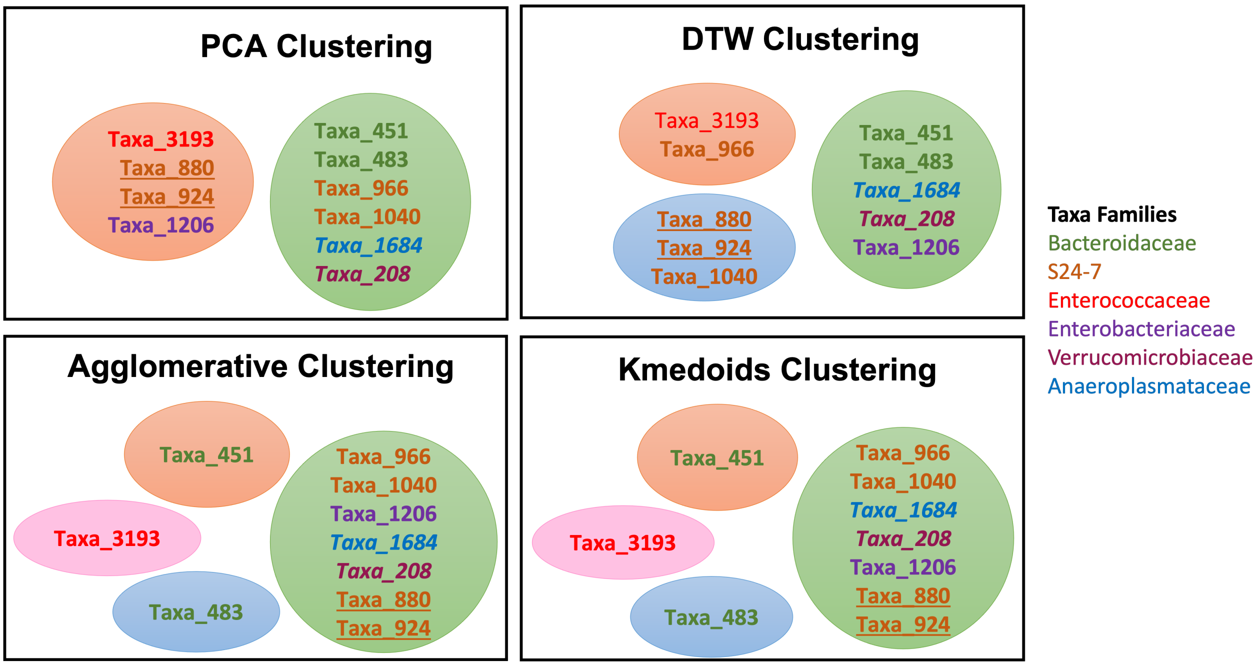

Figure S9: **VREfm study.** Cluster assignment in the VRE phase for the ten most abundant taxa. Colour of the circle reflects the cluster colour from Figure S8. For example, *Enterococcaceae* (Taxa_3193) belong to a pink cluster in agglomerative clustering, shown as cluster 4 in Figure S8 F. Text colour indicates the families the taxa. K-medoids and agglomerative clustering produced identical cluster assignments. The taxa that belongs to S24-7 (*Muribaculaceae*) family tend to cluster together.

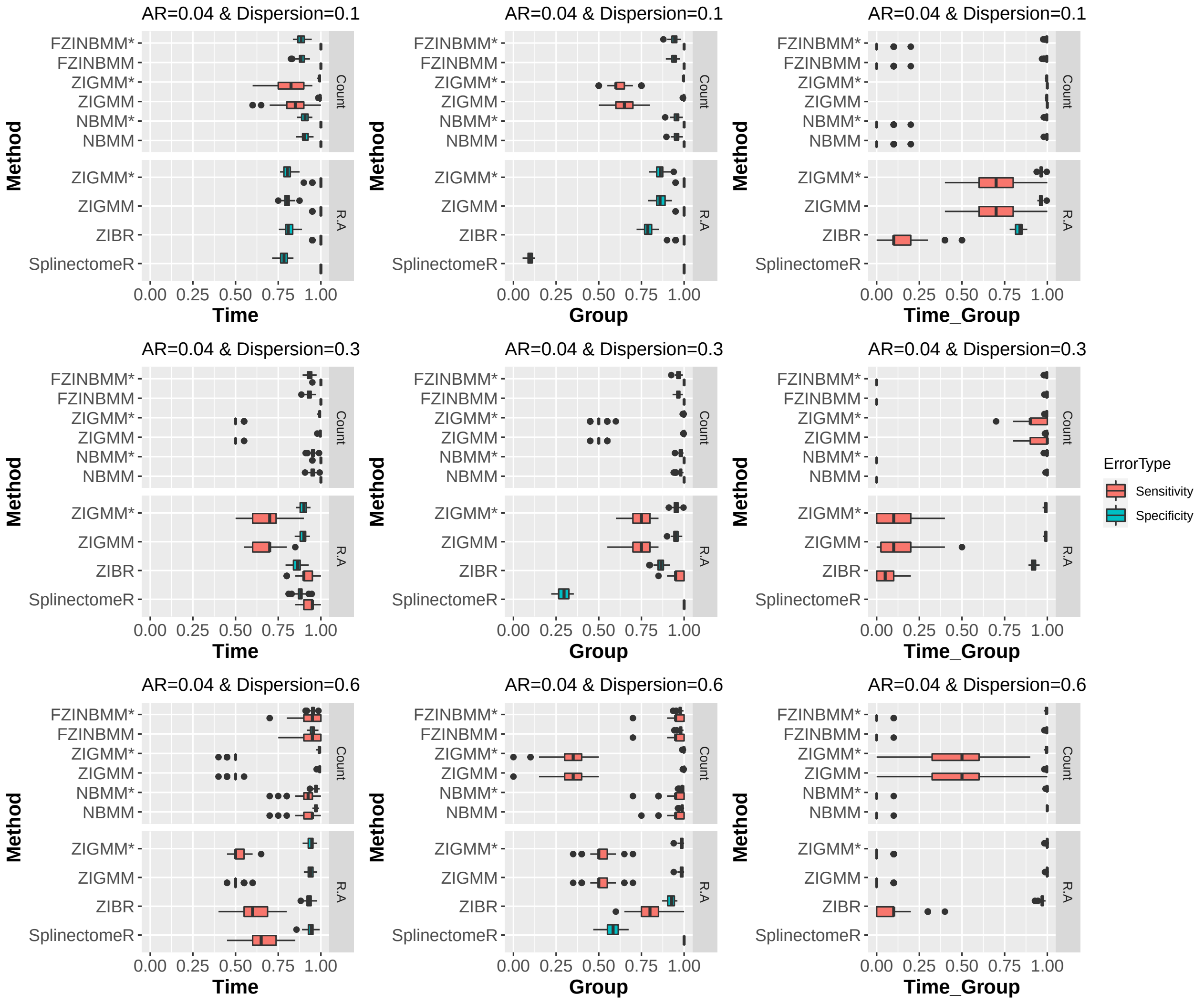

Figure S10: **Simulation study.** Sensitivity and specificity results from different differential abundance analysis for time effect, group effect and time group interaction effect across different dispersion values when AR is equal to 0.04. As the dispersion values are increased, the ability to detect the time effect, group effect and time group interaction effect of all methods are decreased. Since the value of AR parameter is small (0.04), the effect of including an AR within-subject correlation into the model (i.e, model names with '$*$') does not improve the results.

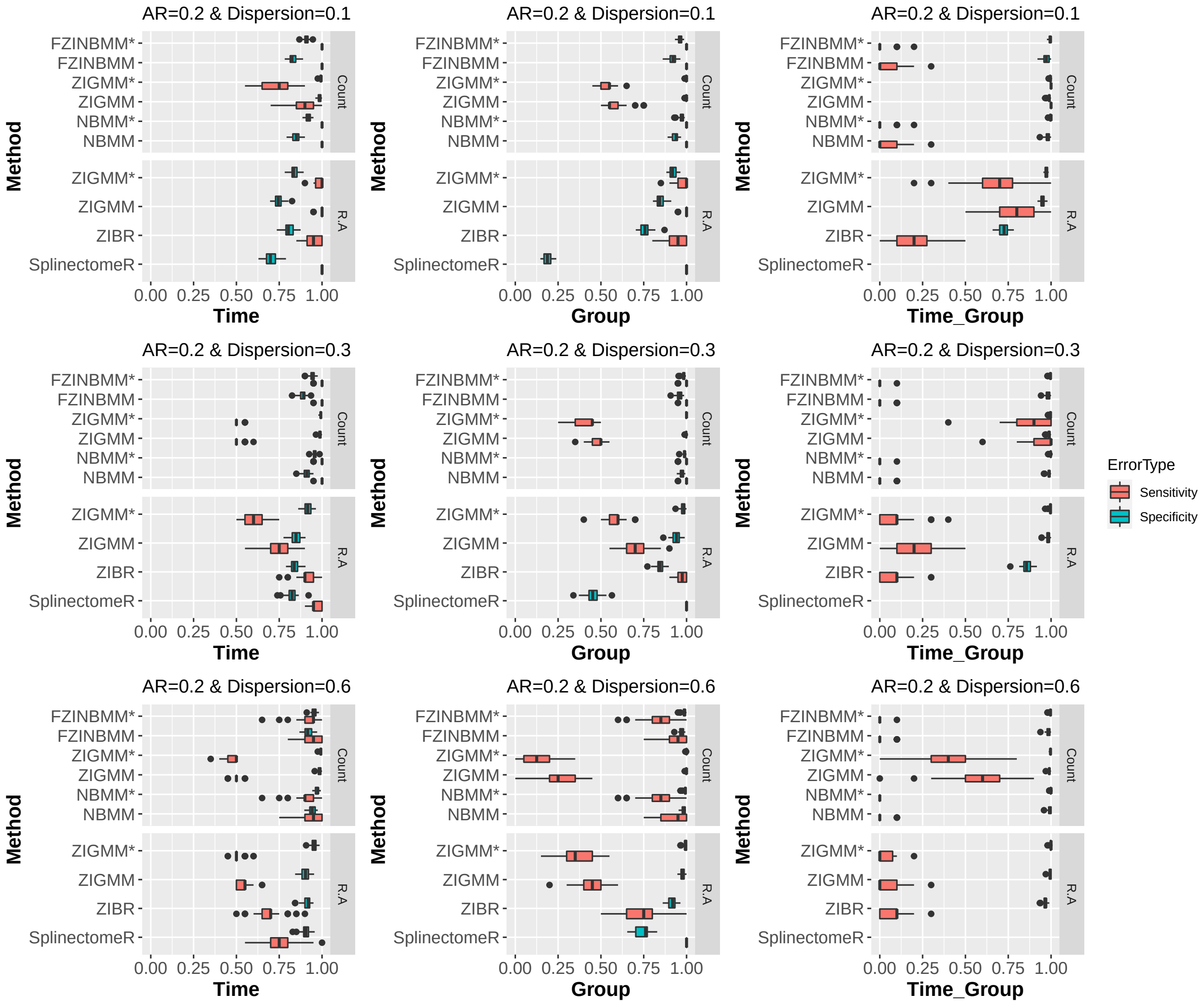

Figure S11: **Simulation study.** Sensitivity and specificity results from different differential abundance analysis for time effect, group effect and time group interaction effect across different dispersion values when AR is equal to 0.2. Similar to Figure S10, the sensitivity results for time effect, group effect and time group interaction effect are high when the dispersion is low. In contrast to Figure S10, there is an improvement in the specificity values for the models with AR within-subject correlation structure compared to the same model without any within-subject correlation.

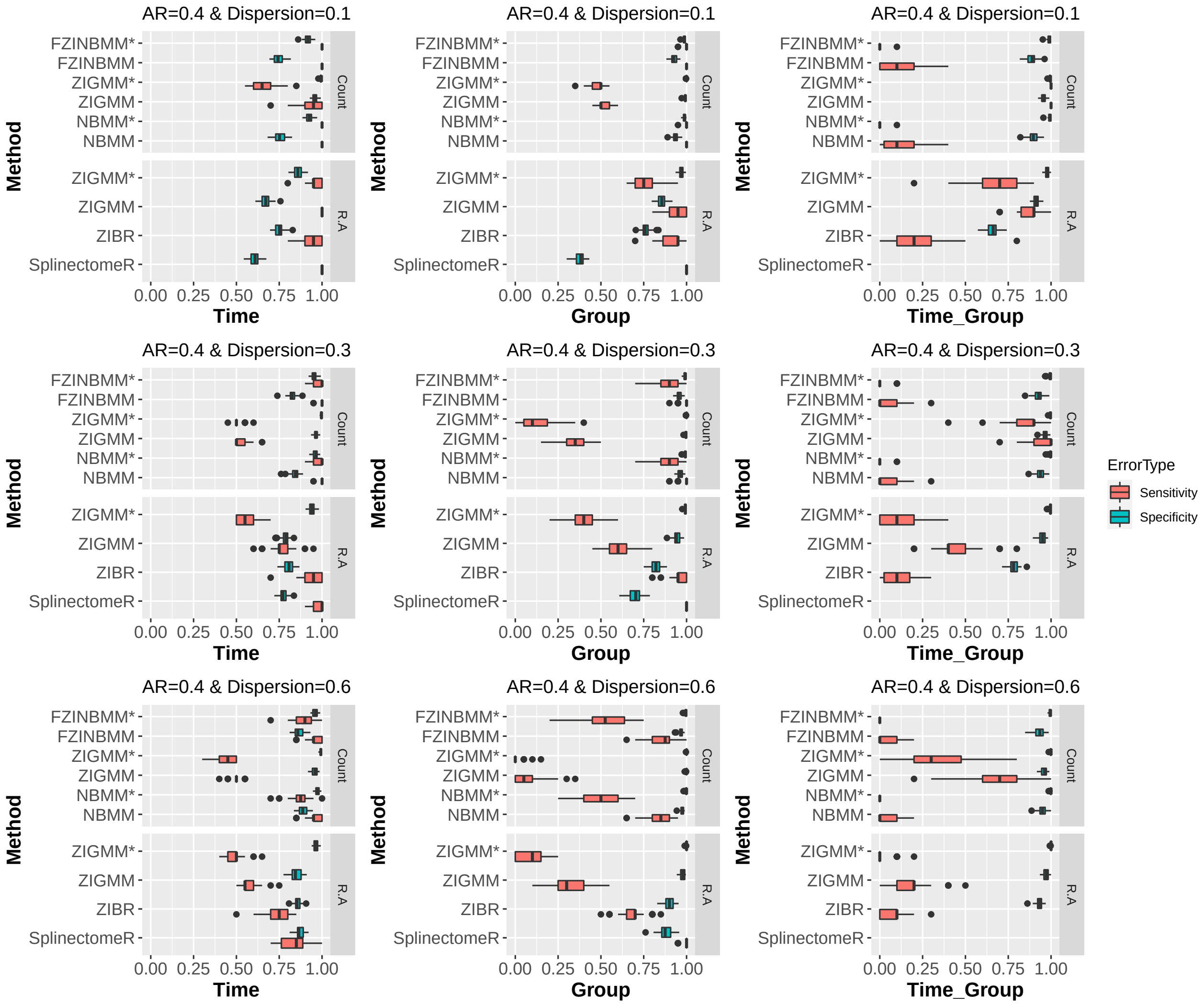

Figure S12: **Simulation study.** Sensitivity and specificity results from different differential abundance analysis for time effect, group effect and time group interaction effect across different dispersion values when AR is equal to 0.4. Similar to Figures S10 and S11, the sensitvity results are highest when the dispersion is low. Also, similar to Figure S11, the models with AR within-subject correlation structure had higher specificity values compared to the same model without any within-subject correlation.

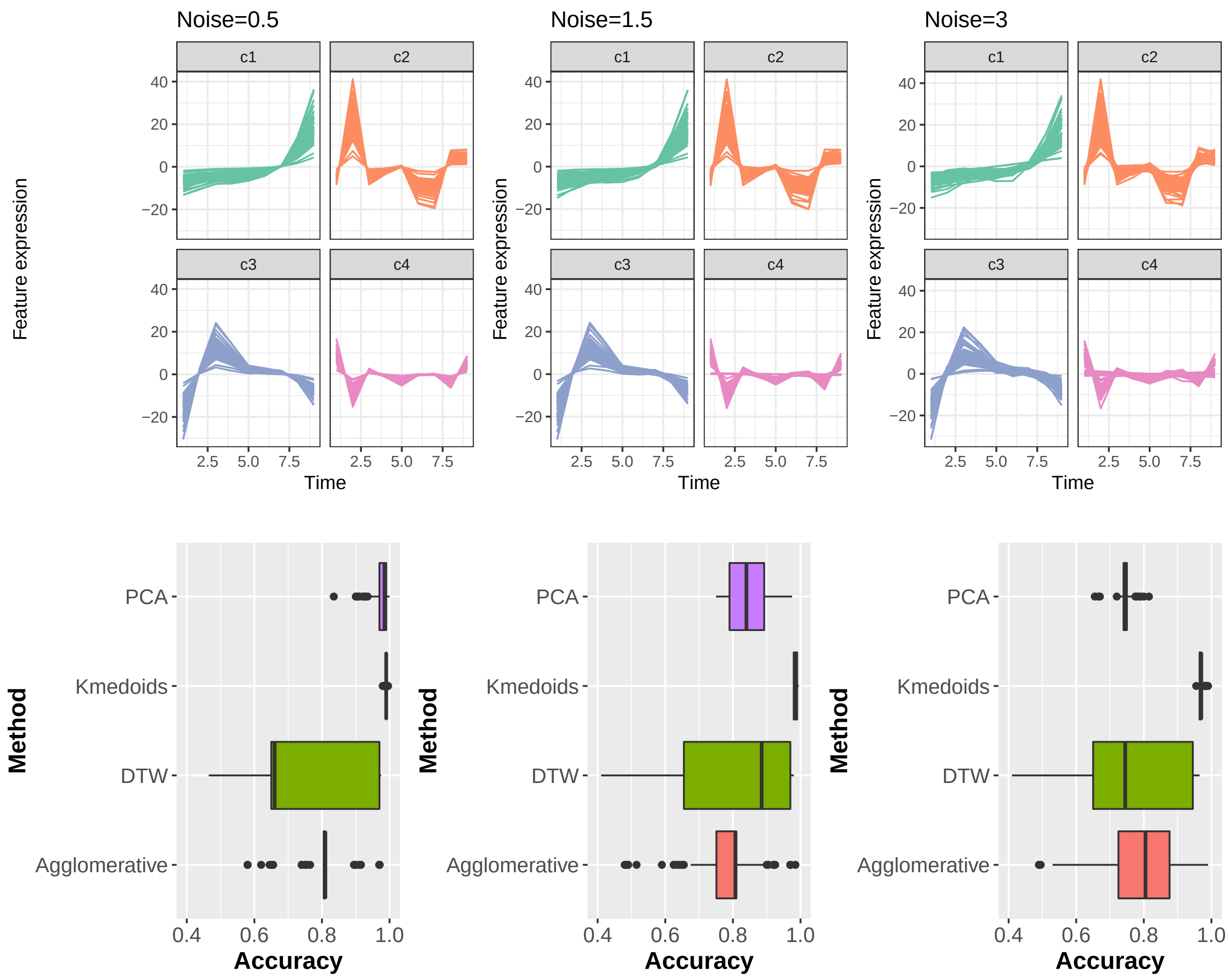

Figure S13: **Simulation study.** Clustering accuracy for PCA, K-medoid, DTW and Agglomerative clustering for centered LMMS profiles. The top panel provides an example of the time profiles for different noise levels with centering. Both k-mediod and PCA clustering perform well when the noise level was low, however, k-mediod outperformed PCA clustering when the noise is increased from 0.5.

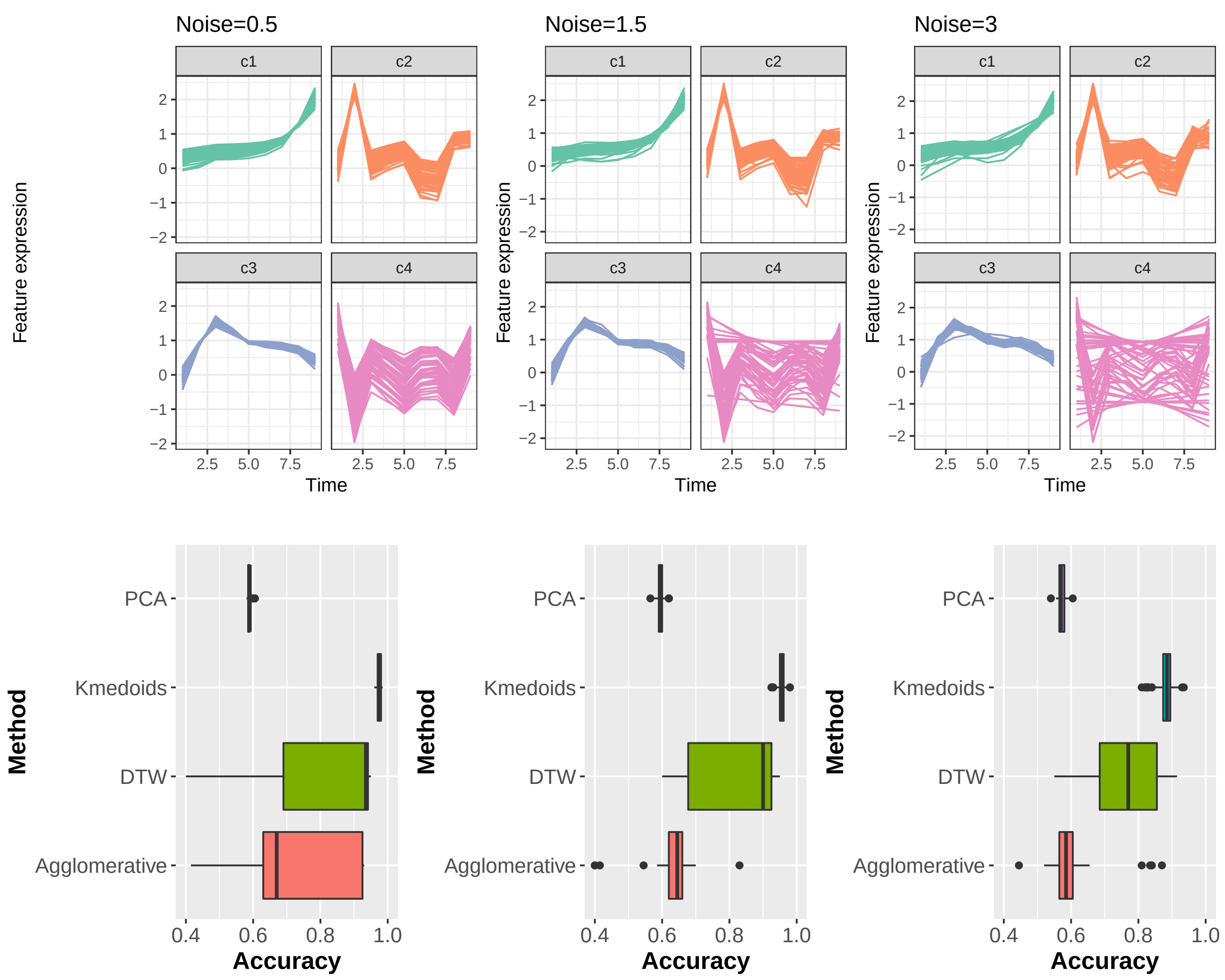

Figure S14: **Simulation study.** Clustering accuracy for PCA, K-medoid, DTW and Agglomerative clustering for scaled LMMS profiles. The top panel provides an example of the time profiles for different noise levels with scaling. For scaled LMMS profiles k-mediod outperform all other clustering methods.

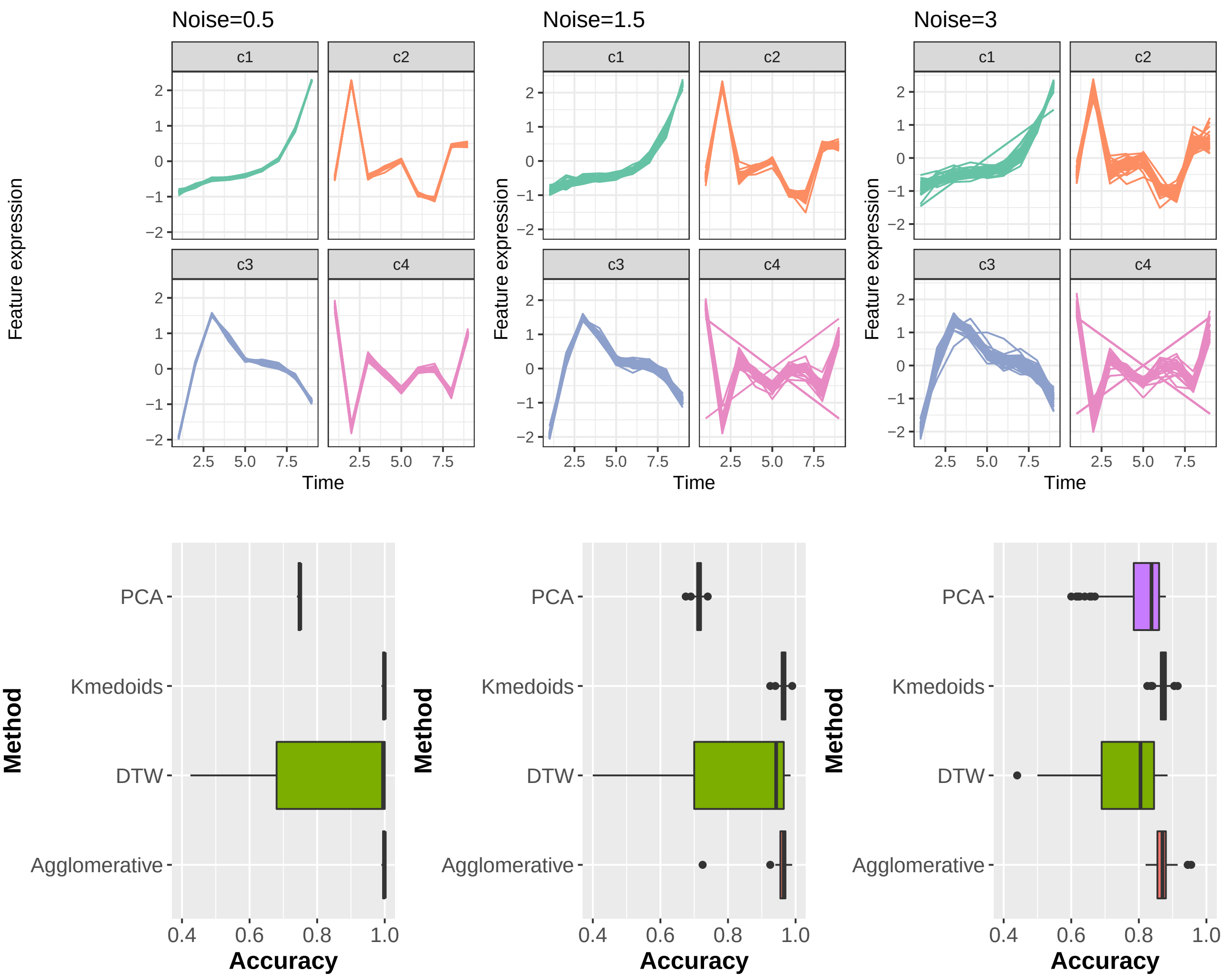

Figure S15: **Simulation study.** Clustering accuracy for PCA, K-medoid, DTW and Agglomerative clustering for centered and scaled LMMS profiles. The top panel provides an example of the time profiles for different noise levels with centering and scaling. For noise levels 0.5 and 1.5, except for PCA clustering all other clustering methods have a similar median accuracy value and for noise level 3, all clustering methods have a similar accuracy.

References

[1] Antoine Bodein, Olivier Chapleur, Arnaud Droit, and Kim-Anh Lê Cao. A generic multivariate framework for the integration of microbiome longitudinal studies with other data types. *Frontiers in genetics*, 10:963, 2019.

[2] Jake R Conway, Alexander Lex, and Nils Gehlenborg. UpSetR: an R package for the visualization of intersecting sets and their properties. *Bioinformatics*, 33(18):2938–2940, 06 2017.

[3] Daniel B DiGiulio, Benjamin J Callahan, Paul J McMurdie, Elizabeth K Costello, Deirdre J Lyell, Anna Robaczewska, Christine L Sun, Daniela SA Goltsman, Ronald J Wong, Gary Shaw, et al. Temporal and spatial variation of the human microbiota during pregnancy. *Proceedings of the National Academy of Sciences*, 112(35):11060–11065, 2015.

[4] Tobias Liboschik, Konstantinos Fokianos, and Roland Fried. tscount: An r package for

analysis of count time series following generalized linear models. *Journal of Statistical Software*, 82(1):1–51, 2017.

[5] Andre Mu, Glen P Carter, Lucy Li, Nicole S Isles, Alison F Vrbanac, James T Morton, Alan K Jarmusch, David P De Souza, Vinod K Narayana, Komal Kanojia, et al. Microbe-metabolite associations linked to the rebounding murine gut microbiome postcolonization with vancomycin-resistant enterococcus faecium. *Msystems*, 5(4):e00452–20, 2019.
